# Human pluripotent stem cell modeling of alveolar type 2 cell dysfunction caused by ABCA3 mutations

**DOI:** 10.1172/JCI164274

**Published:** 2024-01-16

**Authors:** Yuliang L. Sun, Erin E. Hennessey, Hillary Heins, Ping Yang, Carlos Villacorta-Martin, Julian Kwan, Krithi Gopalan, Marianne James, Andrew Emili, F. Sessions Cole, Jennifer A. Wambach, Darrell N. Kotton

**Affiliations:** 1 Center for Regenerative Medicine of Boston University and Boston Medical Center, Boston, Massachusetts, USA.; 2 The Pulmonary Center and Department of Medicine, Boston University School of Medicine, Boston, Massachusetts, USA.; 3 Division of Newborn Medicine, Edward Mallinckrodt Department of Pediatrics, Washington University School of Medicine and St. Louis Children’s Hospital, St. Louis, Missouri, USA.; 4 Departments of Biology and Biochemistry, Boston University School of Medicine, Boston, Massachusetts, USA.; 5 University of Massachusetts Chan Medical School, Worcester, Massachusetts, USA.

**Keywords:** Cell Biology, Stem cells, Genetic diseases, Human stem cells, iPS cells

## Abstract

Mutations in ATP-binding cassette A3 (ABCA3), a phospholipid transporter critical for surfactant homeostasis in pulmonary alveolar type II epithelial cells (AEC2s), are the most common genetic causes of childhood interstitial lung disease (chILD). Treatments for patients with pathological variants of *ABCA3* mutations are limited, in part due to a lack of understanding of disease pathogenesis resulting from an inability to access primary AEC2s from affected children. Here, we report the generation of AEC2s from affected patient induced pluripotent stem cells (iPSCs) carrying homozygous versions of multiple *ABCA3* mutations. We generated syngeneic CRISPR/Cas9 gene-corrected and uncorrected iPSCs and ABCA3-mutant knockin ABCA3:GFP fusion reporter lines for in vitro disease modeling. We observed an expected decreased capacity for surfactant secretion in ABCA3-mutant iPSC-derived AEC2s (iAEC2s), but we also found an unexpected epithelial-intrinsic aberrant phenotype in mutant iAEC2s, presenting as diminished progenitor potential, increased NFκB signaling, and the production of pro-inflammatory cytokines. The ABCA3:GFP fusion reporter permitted mutant-specific, quantifiable characterization of lamellar body size and ABCA3 protein trafficking, functional features that are perturbed depending on *ABCA3* mutation type. Our disease model provides a platform for understanding ABCA3 mutation–mediated mechanisms of alveolar epithelial cell dysfunction that may trigger chILD pathogenesis.

## Abstract 2

A human pluripotent stem cell-derived model mirrors ABCA3 mutation-mediated alveolar type 2 cell dysfunction.

## Introduction

Childhood interstitial lung disease (chILD) is a heterogenous group of genetic lung disorders affecting infants and children who typically present with a range of symptoms and signs such as respiratory distress, hypoxemia, and diffuse pulmonary infiltrates (1–4). While many causal genes have been identified for chILD (5–8), the most common are autosomal recessive mutations in the gene encoding ATP-binding cassette A3 (ABCA3) (1, 3, 9), a lamellar body–associated (LB-associated) phospholipid transporter that is expressed in the lung within alveolar type II epithelial cells (AEC2) (10–12). Bronchoalveolar lavage samples from patients carrying biallelic *ABCA3* mutations have decreased or altered composition of pulmonary surfactant (13), a mixture of approximately 90% phospholipids and 10% proteins that is secreted by AEC2s (14, 15) and reduces surface tension in the alveoli of air breathing organisms. While deficiency in the functionality of ABCA3 lipid transport results in altered phospholipid composition of surfactant and may contribute to disease pathogenesis, *ABCA3* mutation-mediated disruption of AEC2 phenotypes by other cellular pathways has not been systematically evaluated in humans.

To date, progress in understanding chILD pathogenesis has been impeded both by, first, an inability to easily access primary human AEC2s from affected children and, second, limited methods to maintain AEC2s from explanted tissue of affected children. Additionally, compound heterozygous mutant *ABCA3* genotypes with novel or rare (minor allele frequency under 1%) variants in affected children have further complicated attempts to understand mutant-specific disease pathogenesis (16–18). Due to these challenges, prior studies have relied on genetically engineered ABCA3 overexpression models in cancer or immortalized cell lines, such as the human embryonic kidney 293 cell line (HEK293) or the lung adenocarcinoma–derived A549 cell line (18–27), neither of which express surfactant genes, produce functional surfactant, or express the *NKX2-1*–driven gene regulatory network that defines all known endogenous lung epithelia in vivo (28–30). Studies utilizing these heterologous cell lines have classified *ABCA3* mutations into 2 broad subtypes: type 1 mutations resulting in mistrafficking of the mutant ABCA3 protein, or type 2 mutations resulting in decreased ATPase-mediated phospholipid transport (19, 21, 23). However, cell lines engineered for constitutive overexpression of ABCA3 do not recapitulate normal regulation of the endogenous ABCA3-encoding locus, potentially undermining the accuracy of these findings. Methods to establish patient-specific induced pluripotent stem cells (iPSCs), advances in gene-editing technologies, and significant progress in directed differentiation to generate iPSC-derived AEC2s (iAEC2s) — cells that transcriptomically and functionally resemble primary AEC2s (31–33) — have recently enabled human AEC2 disease modeling (34, 35). These edited iPSCs have facilitated functional characterization of the consequences of surfactant processing defects caused by *SFTPB* and *SFTPC* mutations (32, 36), other causes of chILD.

Here, we apply these techniques to study the downstream effects of *ABCA3* mutations in chILD patient-specific iAEC2s as well as in iAEC2s derived from iPSCs engineered to carry identical homozygous mutations by gene editing. Because most patients carry compound heterozygous *ABCA3* mutations, making the study of individual mutations difficult, we focused on identifying infants and children carrying homozygous *ABCA3* type 1 and type 2 mutations along with documented chILD clinical phenotypes. By differentiating these ABCA3 mutant patient iPSC-lines and engineered knock-in iPSCs into iAEC2s in parallel with their gene-corrected syngeneic control lines, we found that patient-specific iAEC2s recapitulate clinically observed surfactant dysfunction through attenuated secretion of surfactant phospholipids and altered lamellar body morphology and function. Unexpectedly, they also exhibit upregulation of a variety of signaling pathways, such as NFκB, in affected iAEC2s, regardless of *ABCA3* mutation type, implying an epithelial-intrinsic aberrant phenotype that results in the secretion of proinflammatory cytokines.

## Results

### E690K and W308R autosomal recessive ABCA3 mutations cause severe respiratory disease in newborns.

**Figure 1:**
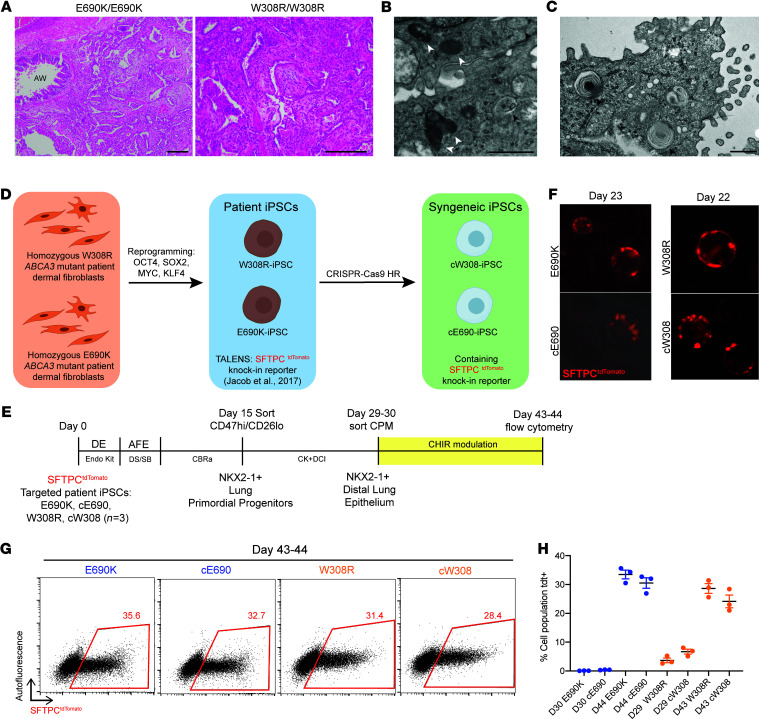
Generation and directed differentiation of patient-specific *ABCA3* mutant and syngeneic gene-corrected iPSC lines produces SFTPC^tdTomato^-expressing iAEC2s. (**A**) H&E staining of lung tissue from each patient of indicated *ABCA3* genotype showing extensive alveolar remodeling, type II cell hyperplasia, interstitial thickening, lymphoid aggregates, and neutrophilic infiltrates (Left), and intraalveolar macrophages (Right). Scale bars: 100 μm. AW, bronchiolar airway. See also Supplemental Figure 1. (**B**) TEM images showing irregular, small lamellar bodies (LBs, black arrow heads) with some abnormal LBs containing dense bodies (white arrow heads) in homozygous E690K *ABCA3* patient lung explant. Scale bar: 1 μm. (**C**) TEM image showing AEC2 containing normal lamellar bodies with loose concentric whorled membranes (black arrow heads) in a patient with pediatric pulmonary hypertension. Scale bar: 600 nm. (**D**) *ABCA3* mutant patient-specific iPSCs reprogrammed from dermal fibroblasts. Using TALENS gene-editing tools, patient-iPSCs are then targeted with SFTPC^tdTomato^ knockin reporter, followed by biallelic gene-correction using CRISPR/Cas9 induced DNA strand break and homologous recombination (HR) to generate syngeneic control iPSC lines. (**E**) Directed differentiation timeline of all patient iPSC lines, biological replicates separated at day 0 (*n* = 3). iPSCs were differentiated as indicated into distal lung epithelium using the protocol described in Jacob et al. (32, 33) with additional CPM sorting followed by CHIR modulation. DE, definitive endoderm; AFE, anterior foregut endoderm. (**F**) Fluorescent images of day 22 to 23 live alveolospheres derived from patient iPSC lines following distal lung differentiation, showing detection of SFTPC^tdTomato^ reporter (red). **G**) Representative flow cytometry analyses of day 43 to 44 cells showing similar frequency of SFTPC^tdTomato^-expressing cells across all lines following CHIR modulation. (**H**) Frequency of SFTPC^tdTomato^-expressing cells from day 29–30 to day 43–44 after CHIR modulation. Biological replicates (*n* = 3). Graphs show mean ± SE. See also Supplemental Figure 2.

To develop an in vitro human model system able to study the downstream consequences of mutations in *ABCA3*, we sought to generate iPSCs from individuals carrying rare homozygous *ABCA3* mutations (Figure 1 and Supplemental Figure 1, A–E; supplemental material available online with this article; https://doi.org/10.1172/JCI164274DS1). We identified 2 individuals, 1 homozygous for ABCA3 W308R (c.922T>C) — which was predicted by in silico algorithms to be a type 1 mutation resulting in disrupted ABCA3 trafficking — and another homozygous for ABCA3 E690K (c.2068G>A) — which was predicted to be a type 2 mutation resulting in disrupted phospholipid transport. Both individuals underwent lung transplantation in the first few years of life, allowing access to lung explant histological analyses, which revealed diffuse AEC2 hyperplasia, alveolar septal thickening, and immune — neutrophilic and lymphoid — infiltrates (Figure 1A, Supplemental Figure 1, A–E, and Supplemental Methods). Ultrastructural examination of the E690K mutant lung explant AEC2s revealed dysmorphic, small, dense lamellar bodies (Figure 1, B and C), as have been reported in prior studies of lung tissue from individuals with biallelic ABCA3 mutations (1).

### Derivation of E690K and W308R ABCA3 mutant and gene-corrected patient iAEC2s.

We reprogrammed dermal fibroblasts procured from these 2 individuals to generate patient-specific iPSC lines from each, carrying either homozygous E690K or homozygous W308R *ABCA3* mutations (hereafter E690K-iPSCs and W308R-iPSCs, respectively) (Figure 1D and Supplemental Figure 1F). Next, using TALENS-mediated monoallelic targeting of the endogenous *SFTPC* locus, we introduced a heterozygous SFTPC^tdTomato^ reporter into each line, as we previously described (32). Because iPSC lines generated from distinct individuals may exhibit different differentiation efficiencies and phenotypes due to differences in genetic backgrounds, we generated syngeneic gene-corrected control iPSCs lines using footprint free CRISPR/Cas9 gene editing to correct both mutant alleles for each corresponding *ABCA3* mutation into WT (corrected lines hereafter referred to as cE690-iPSCs and cW308-iPSCs, Figure 1D and Supplemental Figure 1, G–I).

Differentiation of each patient-specific iPSC line according to our previously published distal lung differentiation protocol (32, 33), followed by serial enrichment of NKX2-1 expressing lung epithelial progenitors using surrogate cell surface markers (Figure 1E and Supplemental Figure 2 and Supplemental Methods[Sec sd]) resulted in the emergence of SFTPC^tdTomato^+ cells in all 4 lines (Figure 1, E–H). After a brief period of CHIR99021 (hereafter referred to as CHIR) withdrawal to mature iAEC2s (Figure 1E and Supplemental Figure 2D) as previously published (33), tdTomato expression was quantified on day 43–44 with no significant differences in reporter expression detected comparing mutant to corrected iAEC2s produced from each line: 33.47% ± 1.53% for E690K cells versus 30.53% ± 1.83% for cE690 cells, and 28.63% ± 1.68% for W308R cells versus 24.13% ± 2.21% in cW308 cells (Figure 1, G and H). Further, there were no statistically significant differences in mRNA expression of a panel of AEC2 markers, *SFTPC, SFTPB, NAPSA, PGC,* and *LPCAT1* between mutant and corrected iAEC2s (FDR > 0.05; *n* = 3 samples per genotype, each purified on day 43–44 based on tdTomato+ sorting and analysis by bulk RNA sequencing; Figure 1, G and H and Supplemental Figure 2, and Supplemental Methods), suggesting no appreciable impact of these ABCA3 mutations on distal lung epithelial–directed differentiation at this stage.

### ABCA3 mutant iAEC2s have attenuated surfactant lipid secretion.

**Figure 2:**
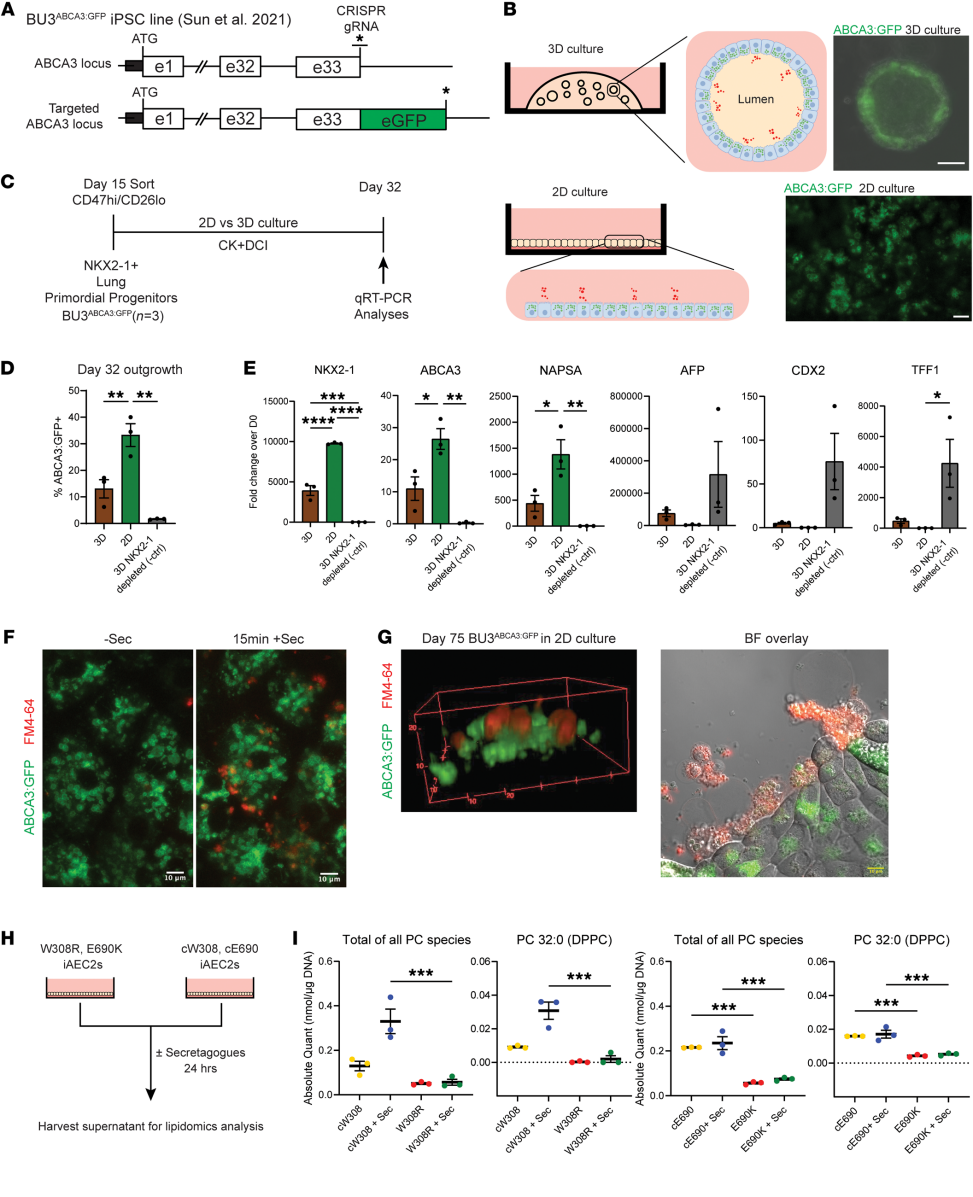
Measurement of secreted surfactant phospholipids in 2D cultures of iAEC2s quantifies surfactant secretion defects resulting from ABCA3 mutations. (**A**) Gene editing generates an ABCA3:GFP fusion reporter as previously published in Sun et al., 2021 (39). *, stop codon. (**B**) Fluorescence microscopy of live iAEC2s in 3D (Scale bar, 50 μm) versus 2D mono-layered cultures (Scale bar, 10 μm). (**C**) Time course of BU3^ABCA3:GFP^ iPSC differentiation for (**D**), *n* = 3 replicates separated at day 0. (**D**) Flow cytometry of ABCA3:GFP expression in day 32 outgrowths of BU3^ABCA3:GFP^ cells enriched for NKX2-1 by CD47^hi^/CD26^lo^ sort on day 15 and grown in 3D or 2D mono-layered cultures, versus negative control (–ctrl; cells depleted in NKX2-1 by sorting on day 15 and then grown in 3D culture). Graph shows mean ± SE, **P ≤ 0.01 by 1-way ANOVA with Tukey’s multiple comparisons test. (**E**) Gene expression (qRT-PCR; 18S normalized fold change in expression compared with iPSCs; 2^–ΔΔCt^ ± SE). *n* = 3 replicates separated on day 0. (**F**) Representative live-cell confocal image of day 75 BU3^ABCA3:GFP^ cells in 2D monolayer culture (left) and after 15 minutes of secretagogue (ATP, PMA) treatment, showing release of surfactant lipids visualized by the FM4-64 lipophilic dye (red). Scale bars: 10 μm. (**G**) 3D rendered z-stack of **F**, 20 minutes after secretagogue treatment shows apical dye released from exocytosing LBs visualized by ABCA3:GFP (green). Scale: 10 μm per tick (left). Brightfield (BF) overlay of surfactant lipid secretion (red) in 2D culture after secretagogue treatment (right). (**H**) Experimental plan for harvesting apical supernatants of 2D cultured patient and gene-corrected syngeneic iAEC2s. (**I**) Levels of total PC species and surfactant specific dipalmitoyl PC (DPPC, PC32:0) in the culture supernatants of indicated patient iAEC2s. Graphs show mean ± SE, *n* = 3 biological replicates; *P ≤ 0.05, **P ≤ 0.01, ***P ≤ 0.001, ****P ≤ 0.0001 by 1-way ANOVA with Tukey’s multiple comparisons test in panels **E** and **I**.

Clinically, biallelic *ABCA3* mutations are associated with altered surfactant composition in affected infants and children, evident as decreased levels in bronchoalveolar lavage fluid of surfactant phospholipid species such as phosphatidylcholine (PC) and dipalmitoyl PC (DPPC, PC32:0) (13, 37, 38). Hence, we sought to measure levels of surfactant phospholipids secreted by the iAEC2s generated from each iPSC line. Because our distal lung cultures described previously (32, 33) are typically conducted in a 3D format where iAEC2s orient apically internally toward each sphere lumen, it is difficult to readily access and measure surfactant secretion. To better access secretion from iAEC2s, we converted 3D cultured iAEC2 spheres into a 2D iAEC2 monolayer culture system, simultaneously enabling easy access to apically secreted material and monitoring of ABCA3 expression in real time by high-resolution imaging (Figure 2, A–C). To determine the impact of a submerged 2D culture on maintenance of iAEC2 identity, including ABCA3 expression, we utilized a normal iPSC line (BU3) engineered with CRISPR/Cas9 gene editing to carry a biallelic ABCA3:GFP knock-in fusion reporter cassette, targeted to the endogenous *ABCA3* locus at the endogenous stop codon (Figure 2A). We recently published complete characterization of this iPSC line (39), hereafter referred to as BU3^ABCA3:GFP^, demonstrating sensitive and specific GFP-based readouts of: (a) *ABCA3* locus activity, (b) expression and intracellular localization of ABCA3 protein via fluorescence microscopic imaging of the ABCA3:GFP fusion protein, and (c) the formation and localization of lamellar bodies in iAEC2s through GFP labelling of the outer limiting membrane of these organelles. To determine whether this line could be adapted to 2D culture (Figure 2B) for real-time imaging of surfactant secretion, which is known to occur through regulated exocytosis of lamellar bodies at the AEC2 apical membrane (40), we differentiated BU3^ABCA3:GFP^ iPSCs into primordial NKX2-1+ lung progenitors and purified them by CD47/CD26 sorting, using methods we previously published (Figure 2C) (33, 41). After transferring sorted progenitors into 2D monolayered versus 3D sphere culture conditions from day 15–32 (Figure 2B), we observed that 2D culture, compared with 3D culture, resulted in brighter and significantly more frequent expression of the ABCA3:GFP reporter (33.27% ± 4.29% versus 13.04% ± 3.43%, respectively) and augmented mRNA expression of *NKX2-1*, *ABCA3*, and *NAPSA*, while minimizing mRNA expression of nonlung endoderm genes *CDX*, *AFP*, or *TFF1*, consistent with augmented iAEC2 differentiation in 2D conditions (Figure 2, A–E). We also observed that iAEC2s could be similarly transitioned from 3D to 2D cultures at multiple time points during iAEC2 epithelial sphere passaging (such as day 75; see below) with successful maintenance or augmentation of ABCA3 expression, suggesting that 2D culture adaptation for distal iPSC-derived lung epithelia is feasible at a variety of time points or developmental stages. Thus, our data indicate that 2D monolayer–cultured cells retain their AEC2 programs and readily express ABCA3 proteins, consistent with our recent report detailing methods for adapting iAEC2s to 2D and air-liquid interface culture conditions (42).

We next sought to determine whether 2D cultured iAEC2s are capable of surfactant phospholipid secretion through regulated ATP-stimulated lamellar body exocytosis, as is known to regulate secretion from AEC2s in vivo. We plated day 75 ABCA3:GFP+ iAEC2s in our 2D culture condition and tested responses to treatment with secretagogues (ATP, 100 μM; Phorbol 12-myristate 13-acetaten, 300 nM) previously shown to result in exocytosis of LB contents in cultured primary AEC2s (43). We monitored potential secretagogue-induced LB exocytosis by culturing cells in a medium containing FM4-64, a lipophilic dye, for live-cell confocal imaging. Within 15 minutes of secretagogue addition, FM4-64 fluorescent signal arose from the GFP+ intracellular vesicles upon their fusion with the apical cell membrane, whereas no signal was detected in the absence of secretagogues (Figure 2, F and G). Thus, ABCA3:GFP+ iAEC2s readily respond to secretagogue signals resulting in the exocytosis of lipophilic contents from within the LB through the apical surface of the cell, into the culture supernatants, resembling the physiological secretion of surfactant from primary AEC2s into alveolar lumens.

Having developed a 2D system able to monitor apical surfactant secretion from iAEC2s, next we assessed whether iAEC2s derived from our patient-specific iPSCs harboring ABCA3 mutations have attenuated surfactant secretion. We performed secretagogue-induced lipidomic analysis of apical supernatants from patient and syngeneic gene-corrected iAEC2s plated in our 2D culture system (Figure 2H). Consistent with previously published clinical findings of decreased PC measured in the bronchoalveolar lavage fluid samples of patients with biallelic *ABCA3* mutations, we observed attenuated secretion of overall PC species, including surfactant-specific DPPC (also PC 32:0), in ABCA3 mutant iAEC2s (W308R and E690K), both at baseline and after secretagogue treatment, compared with their syngeneic gene-corrected control iAEC2s (cW308 and cE690, respectively, Figure 2, H and I). While E690 corrected (cE690) iAEC2s did not have as robust a response to secretagogues as corrected W308 (cW308) cells, potentially due to the slightly higher baseline (presecretagogue) secretion from cE690 cells, regardless, ABCA3 gene correction resulted in higher secretion in both mutant iAEC2 genotypes, both at baseline and after secretagogue exposure (Figure 2I). These results indicate that reduced surfactant secretion in these patients resulted from the *ABCA3* mutations and demonstrate the feasibility of functional restoration of ABCA3-dependent surfactant lipid transport through *ABCA3* gene editing. Further, we conclude that the surfactant dysfunction phenotype observed in these patients is captured by our in vitro patient-specific iPSC-derived iAEC2 disease model.

### iAEC2s expressing E690K and W308R mutant ABCA3 proteins contain smaller lamellar bodies.

**Figure 3:**
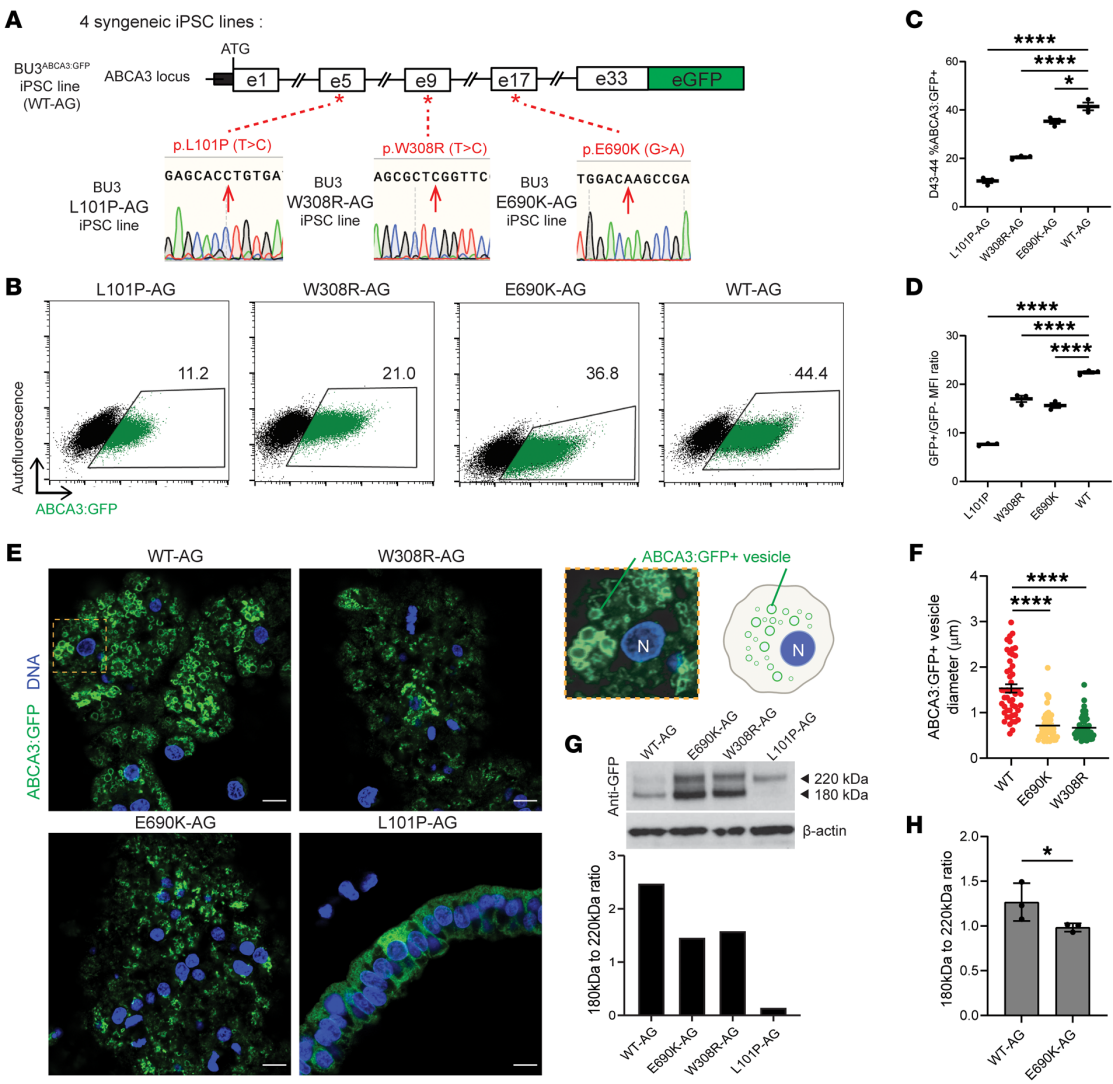
ABCA3 protein mistrafficking/misprocessing and reduced lamellar body size results from subtypes of ABCA3 mutations in human iAEC2s. (**A**) Biallelic editing of the endogenous ABCA3 locus to engineer an ABCA3:GFP fusion reporter in the BU3^ABCA3:GFP^ iPSC line. Three new separate syngeneic mutant iPSC lines were then generated by biallelic editing of the parent line at locations indicated by each * corresponding to either L101P, E690K, or W308R ABCA3 missense mutations. (**B**) Flow cytometry of day 43 to 44 cells after distal lung differentiation of indicated lines, quantifying expression of indicated ABCA3:GFP (AG) proteins. (**B** and **C**) GFP+ cell percentages; (**D**) mean fluorescence intensity (MFI of GFP positive/GFP negative cells). Bars represent mean ± SE (*n* = 3 replicates separated at day 0). (**E**) Confocal microscopy of iAEC2s expressing either WT or each indicated mutant ABCA3:GFP (AG) fusion protein (green). Nuclei (blue). Scale bars: 10 μm. Magnification of WT-AG (orange box) alongside a cartoon showing nucleus (N) and intracellular vesicles outlined by ABCA3:GFP (right). NB: the L101P-AG panel shows diffuse cytoplasmic GFP appearance (protein mistrafficking); there is no reproducible cell morphology change resulting from L101P-AG. (**F**) Diameter of GFP+ intracellular vesicles in indicated iAEC2s. *n* = 50 vesicles per genotype in 8–10 cells/genotype. Graph shows mean ± SE. *P ≤ 0.05, ****P ≤ 0.0001 by 1-way ANOVA with Tukey’s multiple comparisons test for panels **C**, **D**, and **F**. (**G**) Western blot immunostained for GFP (or housekeeping protein, β-actin) to assess processing of the 220 kDa to 180 kDa ABCA3:GFP fusion protein and its cleavage product. Syngeneic iAEC2s were generated from either WT BU3^ABCA3:GFP^ iPSCs or the indicated mutant lines. (**H**) Ratios of 180 kDa to 220 kDa ABCA3 cleavage from **G**. Graph shows mean ± SE. *P ≤ 0.05 by unpaired, 1-tailed Student’s *t* test (*n* = 3).

**Figure 4:**
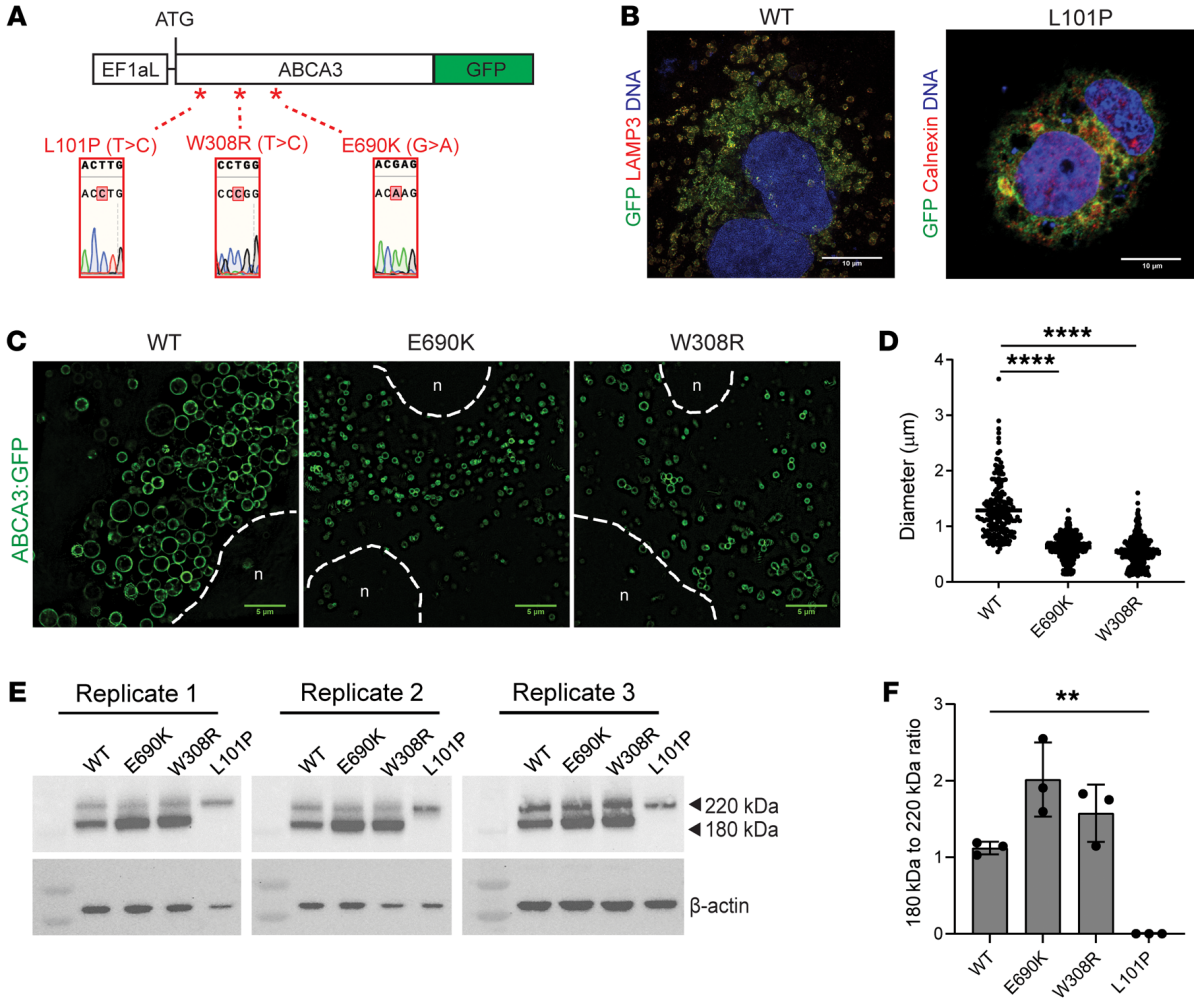
Protein trafficking and lamellar body phenotypes are recapitulated in response to lentiviral forced overexpression of mutant and normal ABCA3:GFP fusion proteins in A549 cell lines. (**A**) Schematic showing locations and sequencing confirmation of 3 introduced ABCA3 mutations in 3 separate lentiviral vectors (each vector contains no or only 1 mutation, denoted by *) used to transduce A549 cells. Each of the 4 lentiviral vectors expressing WT or mutant ABCA3:GFP fusion constructs were driven by a constitutively active promoter EF1aL. *, Site directed mutagenesis of missense mutations confirmed by sequencing. (**B**) Representative confocal fluorescence microscopy of A549 cells expressing WT and L101P ABCA3:GFP fusion proteins (green) costained with lamellar body marker LAMP3 (red, left) and ER marker calnexin (red, right). Nuclei (blue). Scale bars: 10 μm. (**C**) Representative live-cell high resolution confocal images showing smaller GFP+ intracellular vesicles formed in cells expressing E690K or W308R mutant ABCA3:GFP fusion proteins compared with WT fusion protein. Nucleus (n), dotted lines. Scale bars: 5 μm. (**D**) Quantitation of the diameter of GFP+ intracellular vesicles in indicated A549 samples. ****P ≤ 0.0001 by 1-way ANOVA with Tukey’s multiple comparisons test. (**E**) Western blot using antibody against GFP to compare 220 kDa to 180 kDa protein cleavage of WT versus E690K, W308R, and L101P mutant ABCA3:GFP fusion proteins over 3 replicates. (**F**) Quantification of percentage of total ABCA3:GFP protein cleaved in each indicated sample. **P ≤ 0.01 by 1-way ANOVA with Tukey’s multiple comparisons test.

We next sought to determine whether ABCA3 mutations are sufficient to result in either ABCA3 protein mistrafficking or altered LB size and function if expressed in iAEC2s of an independent genetic background. We thus performed CRISPR/Cas9–based biallelic single nucleotide mutagenesis of the ABCA3 locus to introduce each *ABCA3* mutation (E690K and W308R) into a normal BU3^ABCA3:GFP^ line, enabling real-time visualization of mutant ABCA3:GFP protein trafficking. To see how these mutations of interest compare against a known, previously characterized, severe mistrafficking (type 1) *ABCA3* mutant (L101P) (19, 21, 23), we also engineered a syngeneic line to carry L101P ABCA3:GFP. Thus, in total, we generated 4 parallel syngeneic iPSC lines able to express WT or the 3 mutagenized variants of ABCA3:GFP fusion proteins from the endogenous human *ABCA3* locus upon differentiation to iAEC2s (henceforth referred to as WT-AG, E690K-AG, W308R-AG, and L101P-AG, Figure 3A). To compare A549 cell lines, which have been published in the past to study ABCA3 biology, to our iAEC2 model, we also generated 4 A549 cell lines using lentiviral constructs similarly encoding GFP fused to the WT, E690K, W308R, or L101P ABCA3 mutant coding sequences. Each lentiviral ABCA3:GFP fusion cassette was driven by a constitutively and ubiquitously active EF1aL promoter (44), resulting in overexpression of these ABCA3:GFP variants in A549 cells (Figure 4A).

We differentiated all 4 BU3 ABCA3:GFP iPSC lines to iAEC2s using our distal lung differentiation protocol (Figure 1E). We observed decreased frequency and intensity of ABCA3:GFP expression in iAEC2s at day 43–44 across all 3 mutant lines compared with the syngeneic WT line (Figure 3, B–D), indicative of possibly lower levels of ABCA3:GFP intracellular protein or incomplete protein folding or trafficking as a direct or indirect result of *ABCA3* mutations.

By harnessing the utility of the ABCA3:GFP fusion reporter construct, we next sought to study whether mutant ABCA3:GFP protein trafficking and LB morphology were affected within either iAEC2s or A549 cells expressing identical mutant ABCA3:GFP proteins. Using high resolution confocal imaging, we first saw an expected vesicular localization pattern of the WT ABCA3:GFP fusion proteins in both the iAEC2 and A549 cells, consistent with our prior profiles of this reporter system (39). We also saw mislocalization of L101P mutant protein, seen intracellularly in a nonvesicular, diffuse cytoplasmic pattern in both iAEC2s and A549 cells (Figure 3E and Figure 4B), confirming the severe type 1 mutant-specific ABCA3 protein mistrafficking phenotype previously only demonstrated in heterologous cell line models (12, 20, 23). Similar to the WT protein, both the E690K and W308R mutant proteins appeared to localize to intracellular vesicles in the iAEC2 and A549 cell models (Figure 3E and Figure 4C). However, vesicles outlined by mutant ABCA3:GFP proteins were significantly smaller in size compared with those outlined by WT proteins with average diameters of 0.72 μm ± 0.04 for the E690K mutant and 0.67 μm ± 0.04 for the W308R mutant compared with 1.53 μm ± 0.09 in WT vesicles (Figure 3, E and F and Figure 4, C and D). These smaller ABCA3 mutant LB sizes are consistent with actual electron micrographs from patients with the E690K mutation (Figure 1B) and previously published findings (1, 9, 27, 45) describing small lamellar bodies in patients with *ABCA3* mutations, regardless of mutation type, presumably due to decreased functional phospholipid transport into these vesicles. Despite these significant intracellular staining differences compared with WT control iAEC2s, gross cellular morphologies were similar between WT controls and all 3 mutant genotypes.

To confirm our confocal findings of loss of function but seemingly intact ABAC3 trafficking in both E690K and W308R mutants, we performed Western blot analysis of intracellular protein extracts prepared from normal versus mutant iAEC2s generated from each line. Immunoblotting with an anti-GFP antibody identified the various forms of proteolytically processed ABCA3:GFP proteins from a 220 kDa to 180 kDa product, a finding that indicates successful post-Golgi ABCA3 trafficking and processing necessary for subsequent localization to LB precursor vesicles (19, 20). Consistent with our confocal observations, in both iAEC2 and A549 models, we found complete absence of the 180 kDa band in the L101P type 1 mutant, consistent with loss of normal processing and trafficking. We also found presence of the 180 kDa band in the E690K and W308R mutants, qualitatively indicating some degree of successful post-Golgi ABCA3 processing and trafficking (Figure 3, G and H and Figure 4E) in both model systems. In contrast, we detected a quantitative difference between the 2 model systems with a small but statistically significant decrease in mutant E690K protein cleavage compared with WT protein. This decrease was detectable in the iAEC2 model (Figure 3H and Supplemental Figure 5), but not in the A549 model system (Figure 4, E and F).

### Co-IP/MS analyses of WT and mutant ABCA3:GFP proteins suggests potential ABCA3 binding partners.

After seeing the severe mistrafficking phenotype of the L101P mutant ABCA3:GFP protein in our iAEC2 model, we next sought to interrogate whether protein-protein interactions of the WT ABCA3 protein would be disturbed by the most severe L101P mutant ABCA3 protein. We first began by using proteomics to characterize potential interacting protein partners of the WT ABCA3 protein, as previous studies have been limited by lack of access to reliable ABCA3 antibodies for protein pull down. To do so, we performed co-IP using a monoclonal antibody against the GFP portion of the WT ABCA3:GFP fusion protein in iAEC2 cell lysates, followed by trypsinization and mass spectrometry analysis (Supplemental Figure 3A). We identified 20 unique candidate binding partners of WT ABCA3 (Supplemental Figure 3B), classified into proteins that have been reported to facilitate transmembrane protein localization, folding, and vesicle trafficking, such as TMED10, RAB7A, and GIPC1 (46–48), as well as additional proteins that potentially facilitate intracellular vesicle and lamellar body exocytosis, such as the SNARE protein assembly regulator, STXBP2. Moreover, we identified proteins related to solute and phospholipid transporters associated with ABCA3:GFP, such as the glucose transporter SLC2A1 and members of the P4-ATPase phospholipid transporter/flippase complex TMEM30A, TMEM30B, and ATP11A, which are responsible for fine tuning surfactant lipid speciation and content (Supplemental Figure 3B). Finally, ABCA3:GFP preferentially associated with the IL-6 signal transducer, IL6ST, and TRAP, a TNF1-associated chaperone, suggesting possible alternate roles or binding partners for ABCA3.

After characterizing the WT ABCA3 potential interacting partners, we next sought to interrogate the protein binding partners of the severely mistrafficked L101P mutant ABCA3:GFP fusion protein. Some protein-protein interactions were retained — particularly those associated with scaffolding factors such as GIPC1 — at a higher normalized affinity level compared with the WT protein. However, the L101P mutant protein showed significantly attenuated binding affinity for lysosomal protein RAB7A; lipid transporting partners, such as the ATP11A; and the TMEM30A, TMEM30B flippase proteins; as well as the vesicular exocytosis regulator STXBP2. We also found L101P had a unique set of protein-binding signatures consisting of various ER-based chaperone proteins responsible for unfolded protein binding, including protein products such as CCT2, CCT3, CCT5, and CCT6A (Supplemental Figure 3B, C). While these results require further validation of protein binding to ABCA3, the proteomic findings are consistent with our previous immunoblot findings and previous studies for L101P in heterologous cell lines (19, 20, 22, 23, 27), suggesting mislocalization of the mutant protein in the ER.

### Transcriptomic analyses of ABCA3 mutant versus gene-corrected iAEC2s reveal upregulation of inflammatory pathways.

**Figure 5:**
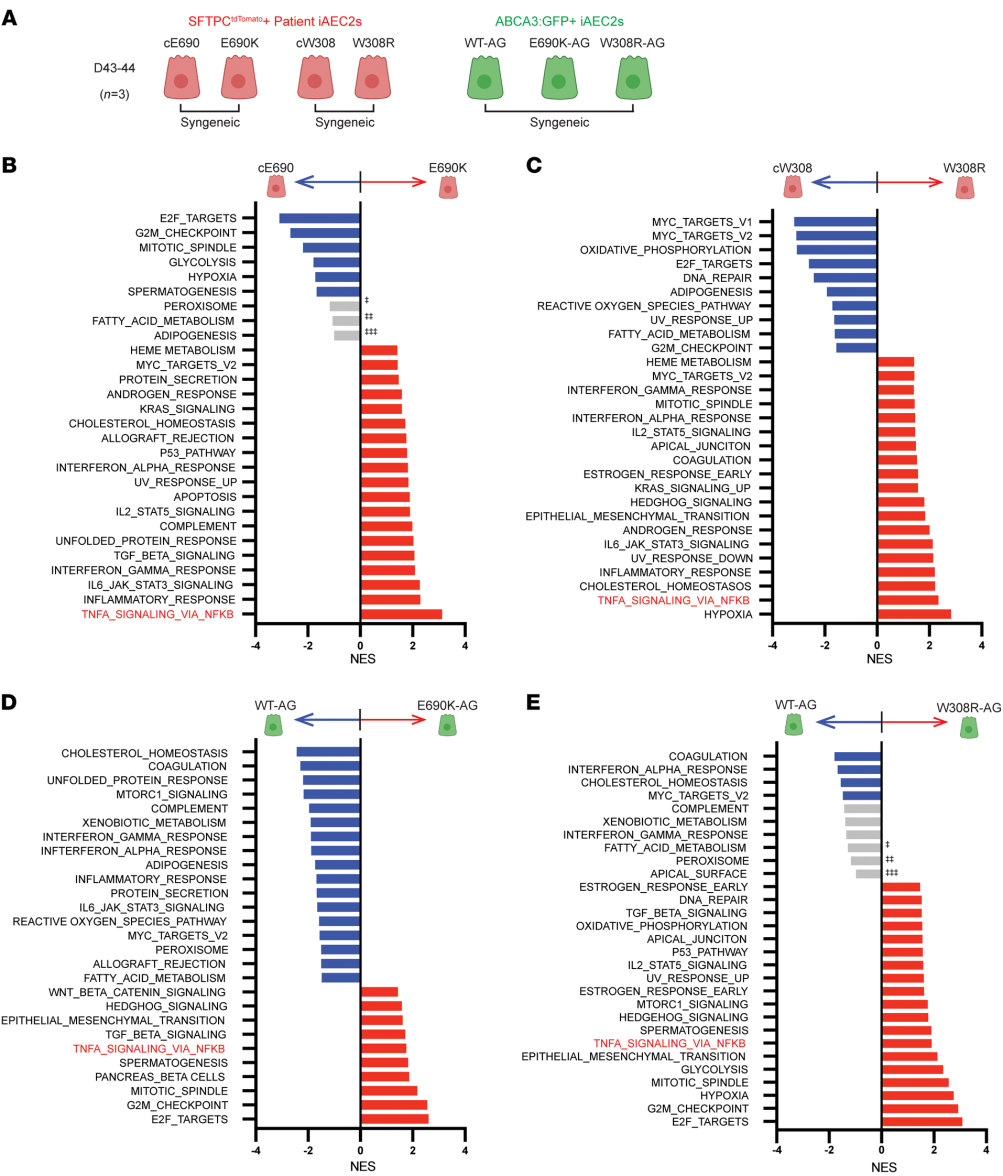
Bulk RNA-Seq of ABCA3 mutant iAEC2s reveals upregulation of inflammatory pathways including the NFκB pathway. (**A**) Schematic for RNA-Seq profiling of sorted, pure populations of day 43–44 SFTPC^tdTomato^+ syngeneic patient iAEC2s versus their gene corrected, paired iAEC2s (E690K, cE690; W308R, cW308 lines from Figure 1) and ABCA3:GFP+ WT (WT-AG) iAEC2s along with their paired CRISPR/Cas9 mutagenized iAEC2s (E690K-AG, W308R-AG from Figure 3). Biological replicates (*n* = 3) were each sorted on the indicated fluorochrome before RNA extraction. (**B**) Bar graph showing normalized enrichment scores (NES) of gene sets enriched in E690K patient iAEC2 (red bars) versus corrected cE690 iAEC2s (blue bars) following GSEA (FDR < 0.05). Gray bars, FDR > 0.05, ^‡^FDR = 0.26. ^‡‡^FDR = 0.46, ^‡‡‡^ FDR = 0.55. (**C**) Bar graph showing NES of gene sets enriched in W308R patient iAEC2s (red bars) versus corrected cW308 iAEC2s (blue bars) following GSEA (FDR < 0.05). (**D**) Bar graph showing NES of gene sets enriched in E690K-AG iAEC2s (red bars) versus WT-AG iAEC2s (blue bars) following GSEA (FDR < 0.05). (**E**) Bar graph showing NES of gene sets enriched in W308R-AG iAEC2s (red bars) versus WT-AG iAEC2s (blue bars) following GSEA (FDR < 0.05). Gray bars, FDR > 0.05. ^‡^FDR = 0.11. ^‡‡^FDR = 0.2, ^‡‡‡^FDR = 0.54.

Although ABCA3 mutations have been assumed to result mainly in defective surfactant secretion, the altered intracellular binding partners suggested by our co-IP studies and known mistrafficking effects also raise the possibility that ABCA3 mutations might also cause generalized perturbations in the AEC2 itself. To provide an unbiased assessment of potential epithelial-intrinsic responses that might result from ABCA3 mutations, we performed transcriptomic analyses comparing mutant to normal or gene-corrected iAEC2s in both the patient-specific genetic backgrounds and in our syngeneic BU3 model system, where we had knocked in the same ABCA3 mutations into normal cells of a distinct genetic background. We performed bulk RNA-Seq on patient-specific SFTPC^tdTomato^+ iAEC2s (E690K and W308R mutants) and their gene-corrected syngeneic control iAEC2s as well as the single WT and 3 CRISPR/Cas9 mutagenized ABCA3:GFP+ iAEC2s (*n* = 3 per genotype × 4 genotypes, day 43–44 of differentiation; Figure 5A).

Pairwise comparisons between each pre- versus post-gene–corrected patient-specific iAEC2 sample revealed 1,384 differentially expressed genes (DEGs) for the E690K mutant and 254 genes for the W308R mutant (FDR < 0.05, absolute log_2_FC > 1, Supplemental Table 1A and B. Among the top 50 genes enriched in the E690K iAEC2s were NFκB signaling factors such as TNFRSF10B, IL23A, and NFKB1, protein chaperones such as HSPA1A, and gene products, such as TGFβ, which have been previously published as playing a role in interstitial lung diseases. Notable top enriched genes in the W308R iAEC2s included known cell death regulators CFLAR and GPC3, and toll like receptor TLR2, possibly suggestive of a diseased cell. Analyses of all DEGs by gene set enrichment analysis (GSEA) using MSigDM Hallmark gene sets (49) revealed inflammatory pathways enriched in the E690K iAEC2s, including TNFα signaling via NFκB, inflammatory response, IL6 JAK STAT3 signaling, interferon responses, complement, IL2 STAT5 signaling, and additional noteable pathways related to TGFβ signaling, the unfolded protein response, and apoptosis (FDR < 0.05, Figure 5B). GSEA analysis of W308R mutant iAEC2 versus corrected cW308R iAEC2s revealed similar enrichment of inflammatory pathways, including TNFα signaling via NFκB, inflammatory response, IL6 JAK STAT3 signaling, IL2 STAT5 signaling, and interferons (Figure 5C, FDR < 0.05). In contrast, proliferative and metabolic pathways such as E2F targets, G2M checkpoint, mitotic spindle, and glycolysis were downregulated (FDR < 0.05) in the E690K mutant cells with proliferative pathways also downregulated in the W308R mutant cells.

Pairwise comparisons between corresponding engineered knock-in mutant and WT ABCA3:GFP+ iAEC2s revealed 1,096 DEGs in the E690K mutant (E690K-AG) and 816 DEGs in the W308R mutant (W308R-AG) compared with the WT iAEC2s (WT-AG, FDR < 0.05, absolute log_2_FC > 1, Supplemental Table 2, A and B). Analyses of DEGs by GSEA revealed enrichment of similar pathways shared with the patient-specific iAEC2 data sets (Figure 5, D and E) including TGFβ signaling, hypoxia, epithelial mesenchymal transition, and IL2_STAT pathways. Consistent with gene-corrected patient iAEC2s, surfactant-related pathways such as adipogenesis, peroxisome, and fatty acid metabolism were differentially regulated in the WT-AG versus mutant cells (Figure 5, D and E). Most notably, the TNFα signaling via NFκB pathway was consistently upregulated across all 4 mutant lines in the 2 distinct genetic backgrounds (both patient-intrinsic iPSC backgrounds and BU3 iPSC knock-in background, Figure 5, B–D).

### Proliferative differences between mutant and gene-corrected iAEC2s.

**Figure 6:**
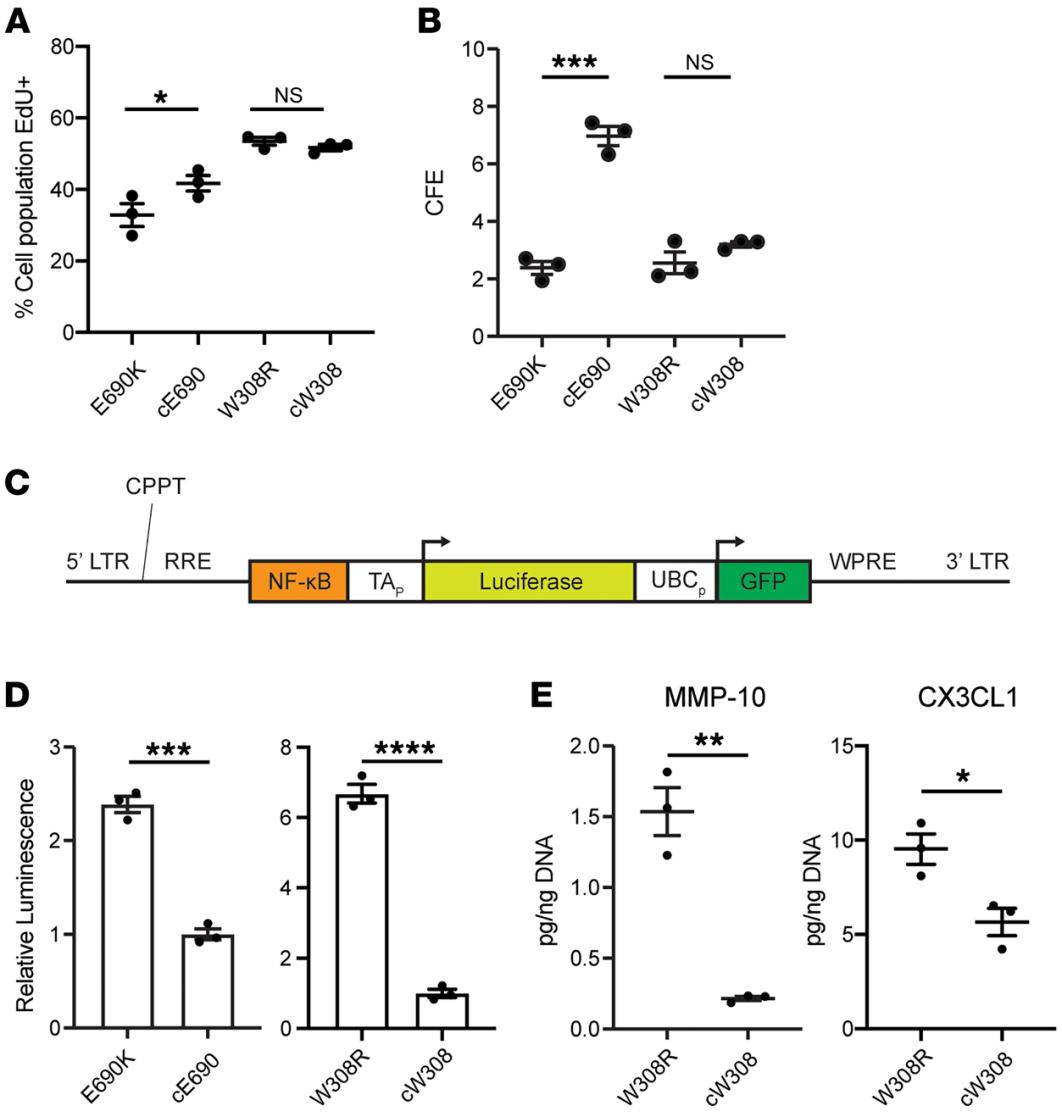
iAEC2s expressing E690K and W308R ABCA3 mutant proteins have increased canonical NFκB signaling activity and variable proliferative capacity. (**A**) Percentages of day 75 patient iAEC2s incorporating EdU after 24 hour incubation. Biological replicates *n* = 3. *P ≤ 0.05, 2-tailed Student’s *t* test. (**B**) CFE reported as percentages of total spheres divided by input cells, of indicated patient iAEC2s on day 57. Biological replicates (*n* = 3), separated at day 0. Bars represent mean ± SE. ***P ≤ 0.001, ns, not significant, 2-tailed Student’s *t* test. (**C**) Lentiviral vector containing 2 gene expression cassettes, one allowing for constitutive expression of GFP for purifying transduced cells, and the second expressing luciferase under the regulatory control of a minimal promoter (TA_p_) adjacent to p50/p65 heterodimer consensus binding sequence (NFκB). This vector (detailed in Alysandratos et al., 2021 [36]) was used to transduce each indicated iAEC2 sample. LTR, lentiviral long terminal repeat; RRE, Rev responsive element; CPPT, central polypurine tract; WPRE, woodchuck hepatitis virus posttranscriptional regulatory element; UBCp, ubiquitin protein-C promoter. (**D**) Relative NFκB pathway activity of lentiviral-transduced (GFP sorted) day 286 W308R and cW308 iAEC2s and day 158 E690K and cE690 iAEC2s measured by levels of D-luciferin luminescence. Replicates (*n* = 3), W308R and cW308 separated at day 148, E690K and cE690 separated at day 0. Graphs show mean ± SE. ***P ≤ 0.001, ****P ≤ 0.0001, 2-tailed Student’s *t* test. (**E**) Levels of indicated cytokines released in the culture supernatants of 2D monolayer cultured patient iAEC2s. Biological replicates (*n* = 3), separated at day 0. Graphs show mean ± SE. *P ≤ 0.05, **P ≤ 0.01, 2-tailed Student’s *t* test.

We functionally assessed whether both *ABCA3* mutations negatively impacted iAEC2 proliferation as predicted by our bulk RNA-Seq results, which showed diminished Myc targets, E2F targets, or cell cycle–associated pathways in the patient-specific mutant iAEC2s (Figure 5). Proliferative capacity is a particularly important characteristic of AEC2s in vivo since they serve as the predominant facultative progenitors tasked with maintaining or replenishing the alveolar epithelium, and diminished AEC2 progenitor and regenerative capacity resulting from ABCA3 deficiency has been suggested in vivo in genetic mouse models (50) but is unstudied in humans. Hence, we performed EdU incorporation assays to formally quantify proliferation in all patient iAEC2 lines. Comparing 24-hour EdU incorporation by SFTPC^tdTomato^+ iAEC2s within each syngeneic iAEC2 paired sample, we found no proliferative difference between W308R and cW308 iAEC2s, but we found lower proliferation in E690K iAEC2s with proliferating cells making up 32.83% ± 3.21% of total cells compared with a higher 41.73% ± 2.20% in the corrected cE690 iAEC2s (Figure 6A). To see whether these proliferative differences functionally translated to changes in clonogenicity, we performed colony forming efficiency (CFE) assays on day 57 epithelial sphere outgrowths from day 43 plated SFTPC^tdTomato^+ patient iAEC2s. Consistent with no differences in the EdU incorporation, we found no CFE changes in the W308R compared with the cW308 iAEC2s, but we observed an approximately 3-fold lower CFE in the E690K mutant compared with cE690 iAEC2s, consistent with reduced progenitor activity (E690K CFE = 2.38% ± 0.23% versus cE690 CFE = 6.97% ± 0.33%; Figure 6B).

### E690K and W308R ABCA3 mutations increase iAEC2 NFκB signaling resulting in secretion of inflammatory cytokines.

Since transcripts related to NFκB signaling were upregulated in common across all ABCA3 mutant iAEC2s regardless of genetic background, we next sought to validate this finding by quantifying the activity of NFκB signaling in iAEC2s. To quantify the level of canonical NFκB pathway activity, we transduced both mutant and syngeneic gene-corrected iAEC2s (*n* = 3) with a lentivirus we have previously published (51) that carries a dual gene expression cassette encoding a constitutively active GFP reporter and a luciferase reporter driven by a minimal promoter with adjacent enhancer consisting of multiple repeats of the p50/p65 heterodimer consensus binding sequence (Figure 6C). Analysis of luciferase activity in GFP+ transduced iAEC2s validated significantly increased NFκB pathway activity in both E690K and W308R mutant iAEC2s (Figure 6D), findings consistent with our prior report of canonical NFκB activation in iAEC2 models of another chILD related mutation, SFTPC^I73T^ (36), and possibly suggesting a common signaling pathway augmented in diseased iAEC2s. Analysis of supernatants from 2D monolayer-cultured W308R mutants versus gene-corrected patient iAEC2s (but not E690K mutants) revealed significantly increased secretion of selected proinflammatory cytokines, such as increased CX3CL1, a potent chemoattractant for lymphocytes and macrophages and a known contributor to the pathogenesis of other ILDs (52–54), and MMP-10, a recently proposed biomarker for idiopathic pulmonary fibrosis (55) (Figure 6E and Supplemental Figure 4), further suggesting that changes in iAEC2s beyond mere surfactant secretion defects, may occur as a result of specific types of *ABCA3* mutations.

## Discussion

Individuals carrying pathological variants of *ABCA3* mainly present with either neonatal respiratory distress (NRDS) in full-term infants or chILD in children, or both. Incidentally, many patients with chILD do not present with NRDS, suggesting surfactant dysfunction alone in neonates is not the primary pathogenic mechanisms triggered by ABCA3 mutations to disrupt AEC2 metabolism for chILD pathogenesis. Thus, current therapies for patients with lung disease due to *ABCA3* mutations are nonspecific and ineffective, with the only known definitive treatment option being lung transplant, as was the case for the children homozygous for E690K or W308R *ABCA3* mutations profiled in our studies (Figure 1, A and B and Supplemental Figure 1I). Development of therapeutic alternatives has been challenging due to an inability to access and culture primary AEC2s from patients with chILD. In the present study, we overcame these hurdles by developing and extensively characterizing an in vitro disease model using iAEC2s derived from patient-specific and ABCA3:GFP reporter–containing iPSC lines, enabling the detailed analysis of *ABCA3* mutant phenotypes compared with syngeneic gene-corrected controls. Through functional, proteomic, lipidomic, and transcriptomic analyses, we find our iAEC2 model recapitulates the clinically observed phenotype of surfactant dysfunction resulting from diminished secretion and predicts a previously unappreciated epithelial-intrinsic aberrant AEC2 phenotype caused by *ABCA3* mutations leading to increased canonical NFκB signaling, and, in some genotypes, to diminished progenitor capacity. Whether this altered cellular phenotype results from a toxic gain of function effect of mutant ABCA3 protein or secondary effects from loss of ABCA3 function will require further research. Our iAEC2 disease model has the potential to advance an understanding of chILD pathogenesis and may provide a foundation for the development of pathway-specific therapeutics designed to reverse the readouts of aberrant iAEC2 function detailed in this report. The introduction of a variety of *ABCA3* mutations into a normal iPSC line resulted in similar perturbations to those observed in patient-specific mutant lines, thus confirming that surfactant dysfunction results from abnormal ABCA3 rather than from associated modifying gene mutations present only in affected patients’ genetic backgrounds.

Applying our model system, we confirmed that, similar to previous studies using HEK293 and A549 cells (19–21, 23), the E690K mutant protein mostly retained WT protein trafficking to LBs, with only a small, though statistically significant, decrease in proteolytic cleavage efficiency compared with WT cells. Unexpectedly, we also found W308R to traffic to LBs similar to WT cells by both fluorescence microscopy and Western blotting, contrary to in silico predictions using sequence-based protein conformational algorithms (56–58). While both mutant proteins retained some degree of proteolytic cleavage and trafficking patterns, we observed smaller LBs formed by the mutant ABCA3 fusion proteins similar to previous studies (27, 45). Combined with measurements of decreased surfactant lipids found in patient iAEC2s, this phenotype further supports our current understanding of ABCA3 as the major surfactant lipid transporter in AEC2s (10–12) and lends support to LB size as a useful readout for small molecule screens to restore ABCA3 function. The finding of smaller lamellar body size in our model is consistent with the abnormally small and dense lamellar body morphologies found in the lung tissues of patients with ABCA3 mutations (Figure 1) (1, 2, 9, 59) and suggests that these smaller lamellar bodies are a direct consequence of dysfunctional mutant ABCA3 rather than the epiphenomena of other perturbations, such as lung infections or ventilator induced lung injuries, which frequently occur in patients with chILD.

Encouragingly, based on similarities between CFTR (ABCC7) — itself an ABC transporter protein (60) and the causal gene for cystic fibrosis (61–63) — and ABCA3, recent studies have repurposed drugs developed to modulate CFTR function for restoration of ABCA3 function in cancer cell line models. These prior studies used readouts of vesicle size and ABCA3 mistrafficking in the context of forced overexpression of mutant ABCA3 proteins in A549 cells (24, 25). While A549 cells — an adenocarcinoma cell line thought to be derived from AEC2s — lack surfactant protein or *NKX2-1* gene expression and thus do not recapitulate normal AEC2 physiology, we were surprised to find that A549 cells do model many of the intracellular features of our iAEC2 mutant ABCA3 model system. For example, when examined head-to-head, A549s overexpressing identical ABCA3 mutant proteins to those expressed from the endogenous locus in our iAEC2s demonstrated qualitatively similar (though not quantitatively identical) protein trafficking phenotypes, along with smaller LBs in E690K and W308R mutants. This finding lends support to the use of A549 cells, in tandem with iAEC2s, for future mechanistic and therapeutic studies. For example, high throughput drug screening could be performed in A549 cells, with potential hits confirmed in a more physiologically relevant cell model, such as iAEC2s or primary AEC2s, if available.

Our ABCA3 disease model leveraging footprint-free CRISPR/Cas9 gene editing tools, enabled the pairwise comparison of syngeneic mutant versus corrected iAEC2s while controlling for the rest of the genetic background. We employed models that carried the patient’s own genetic background, including any genetic susceptibility to the disease, as well as mutagenized WT iPSC lines (BU3) engineered to have the same *ABCA3* mutations, allowing for stringent identification of mutant-specific pathways in both diseased and healthy genetic backgrounds. Transcriptomic analyses by bulk RNA-Seq across all 3 genetic backgrounds (E690K individual, W308R individual, and knockin mutant BU3 iPSC lines) revealed enrichment of a wide range of inflammatory pathways with the NFκB signaling pathway shared across all *ABCA3* mutant iAEC2s (Figure 5). This implied aberrant AEC2 phenotype resulting from *ABCA3* mutations in AEC2s, is consistent with the published conditional mouse ABCA3 knock out model, which showed increased inflammation in mouse lungs in vivo resulting from loss of ABCA3 function (50). Importantly, Rindler et al. also observed that after tamoxifen induced ABCA3 deletion in a portion of AEC2s in these mice, those AEC2s with intact ABCA3 protein appeared to survive, proliferate, and reconstitute the AEC2 compartment with ABCA3 expressing cells, thus implying a survival advantage to AEC2s with normal ABCA3 compared with those lacking intact ABCA3. Interestingly, we also found proliferative pathways enriched in our gene-corrected patient iAEC2s with the cE690 gene-corrected iAEC2s, demonstrating improved proliferation and CFE (Figure 6). A proliferative advantage for AEC2s containing normally functioning ABCA3, if confirmed, may provide the foundation for delivery of gene correcting vectors as a future therapeutic option, as recently demonstrated in mouse lungs following in utero gene-correction of mutant SFTPC^I73T^ by Alapati et al. (64).

An important question to consider is how NFκB signaling is activated in mutant iAEC2s. It is possible that some of the enriched pathways upregulated in mutant iAEC2s are upstream of NFκB signaling, such as the unfolded protein response (UPR) or dysregulation of overall intracellular lipid homeostasis without adequate ABCA3 function. Future studies focused on validation of these potential upstream pathways will likely further inform disease mechanisms. Moreover, our recent report detailing the downstream impact of SFTPC^I73T^ mutations in iAEC2s also revealed upregulation of the NFκB signaling pathway (36). This suggests a potential common response downstream of differing types of perturbed pathways resulting from disparate mutations that alter AEC2 function.

Our model serves as a reductionist, epithelial-only model system, allowing delineation of potential disease activating mechanisms that initiate in the cell type carrying the AEC2-specific mutation of interest, without the presence of additional neighboring lineages that can confound this understanding. On the other hand, a complete understanding of chILD will require more complex models introducing these lineages that likely respond to and interact with the initiating dysfunctional cell type. As a result of increased NFκB pathway activity, we found secretion of proinflammatory cytokines MMP10 and CX3CL1 in the W308R mutant iAEC2s. Future studies with animal models and more physiologically complex culture systems such as those incorporating coculture with relevant immune and mesenchymal cell types, will be needed to facilitate a more comprehensive understanding of the paracrine effects of diseased AEC2s as a result of NFκB upregulation caused by *ABCA3* mutations.

Thus, our study reports a human disease model system based on iAEC2s carrying pathogenic ABCA3 mutations and suggests epithelial-intrinsic contributions to chILD pathogenesis. This model should now facilitate the development of effective targeted therapies able to prevent or reverse AEC2 dysfunction in patients with chILD carrying *ABCA3* mutations.

## Methods

### Patient iPSC derivation and maintenance.

All human iPSCs were maintained in feeder-free conditions, cultured on Matrigel-coated (Corning) plates in mTeSR media (StemCell Technologies), and passaged using Gentle Cell Dissociation Reagent (StemCell Technologies). Reprogramming of the BU3 human iPSC line was previously reported in Kurmann et al. (65), and editing of this line to target an ABCA3:GFP fusion cassette to the endogenous ABCA3 locus (BU3^ABCA3:GFP^) was previously reported in Sun et al. (39). For derivation of ABCA3 mutant patient-specific iPSC lines, dermal fibroblasts from each individual were received from Washington University School of Medicine. Genetic evaluation found no mutations in other genes associated with surfactant production, such as *SFTPC* or *SFTPB* genes. Reprogramming of dermal fibroblasts from individuals with homozygous E690K or homozygous W308R *ABAC3* mutations was performed as detailed in the Supplemental Methods.

### Gene editing of human iPSC lines.

E690K and W308R ABCA3 mutant patient-derived iPSC lines were monoallelically targeted with a tdTomato reporter at the ATG start site of the endogenous SFTPC locus using TALENS gene editing tools following the same methods detailed in Jacob et al. (32). Subsequent biallelic, footprint-free *ABCA3* gene correction of patient iPSC lines by CRISPR/Cas9 gene editing is detailed in the Supplemental Methods. To introduce *ABCA3* mutations and the ABCA3:GFP reporter by knockin to normal BU3 iPSCs, we used CRISPR/Cas9 single nucleotide E690K and W308R mutagenesis of the WT BU3^ABCA3:GFP^ iPSC line, the same guide RNAs used for gene-correction of patient-specific iPSC lines were used in conjunction with new ssODN donors to introduce the mutated sequences, as detailed in the Supplemental Methods. For the L101P mutagenesis of BU3^ABCA3:GFP^ iPSC line, additional gRNA and mutant carrying donor template sequences were employed, as detailed in the supplement.

### Directed differentiation and maintenance of iAEC2s.

Directed differentiation of iAEC2s were performed as detailed in our previously published protocol (32, 33). In brief, day 0 PSCs were differentiated into definitive endoderm (day 0–3) using StemDiff Endoderm Kit (Stem Cell Technologies), followed by anterior foregut endoderm (day 3–6) using DS/SB media (2 μM dorsomorphin, Stemgent; 10 μM SB431543, Biotechne), then further specified into NKX2-1+ lung epithelial progenitors using CBRa media (3 μM CHIR99021, Biotechne; 10 ng/mL rhBMP4, BioTechne; 100 nM retinoic acid, Sigma-Aldrich; day 6–15). On day 15, NKX2-1 expressing lung epithelial progenitors were sorted either by NKX2-1^GFP^(BU3-NGST line) or using CD47^hi^/CD26^lo^ sorting to enrich for NKX2-1+ cells (41). Sorted cells were plated in 3D Matrigel cultures and fed with distalizing CK+DCI medium (3 μM CHIR99021 [Tocris Biosciences], 10 ng/mL KGF [R&D], 50 nM dexamethasone, 0.1 mM cyclic AMP, and 0.1 mM IBMX [all from Sigma-Aldrich]), as detailed in Jacob et al (33).

Additionally, to increase the frequencies of SFTPC^tdTomato^ or ABCA3:GFP-expressing iAEC2s, withdrawal and addback of CHIR99021 to distal lung progenitor cells was conducted as previously published in Jacob et al. (33), first by plating day 30 CPM sorted cells in 3D matrigel and feeding with CK+DCI and RI for 48 hours, followed by refeeding with KGF+ DCI and RI (KDCI+RI; i.e., CHIR withdrawal) for 5 days, followed by refeeding with the standard CK+DCI media for the duration of the experiment indicated in the text.

### 2D monolayer culture of iAEC2s.

2D monolayered iAEC2 cultures were prepared by plating either day 15 CD47^hi^/CD26^lo^ sorted lung progenitors, or day 43+ alveolospheres as indicated in the text, after treating with trypsin to prepare a single-cell suspension. Single-cell suspensions were then plated on Matrigel-coated 48-well tissue culture plates (Corning) at 300,000 to 600,000 cells per well. 2D cultures were fed with CK+DCI with RI every other day until confluent. Quantification of surfactant secretion by mass spectroscopy and gene expression by either RT-qPCR or immunofluorescence microscopy are all detailed in the Supplemental Methods.

### ABCA3:GFP Co-IP and Mass Spectrometry analyses.

To identify potential protein binding partners for ABCA3, we prepared iAEC2 cell pellets as indicated in the text, immunoprecipitated ABCA3:GFP fusion protein using a monoclonal anti-GFP antibody (Invitrogen GF28R) versus IgG control and identified candidate coprecipitated peptides and proteins, by mass spectroscopy (< 1% FDR cutoff) as detailed in the Supplemental Methods.

### Bulk RNA-Seq and bioinformatic analysis.

Triplicate differentiations of each indicated line were performed and RNA extracts of iAEC2s (*n* = 3 per line) profiled by bulk RNA-Seq with bioinformatics analyses, including GSEA, performed as we have previously published in Sun, et al. (39), and as further detailed in the Supplemental Methods. Data sets are available for free download from the gene expression omnibus (https://www.ncbi.nlm.nih.gov/geo/; GSE205319; GSE205318) or through the bioinformatics portal at www.kottonlab.com

### EdU incorporation and CFE assays.

CFE assays and EdU uptake scores after 24 hours of incubation in cultured iAEC2s were both prepared as detailed in the Supplemental Methods.

### Measurement of NFκB pathway activity in patient iAEC2s.

Bioluminescence quantification of p50/65 heterodimer binding activity was performed in AEC2s using our published lentiviral NFκB signaling reporter vector (51) with methods for transduction of iAEC2s, sorting, and bioluminescence measurements detailed in our prior publication (36). Day 258 W308R and cW308 patient iAEC2s, and day 158 E690K and cE690 iAEC2s were transduced for biolumescence profiling. To quantify cytokine secretion, supernatants were harvested from iAEC2s after 2D culture and analyzed through a human magnetic Luminex assay (R&D Systems) on Bio-Plex 200 multiplexing analyzer system (Bio-Rad), with cytokine list and methods detailed in Supplemental Materials.

### Statistics.

Unless otherwise specified in the text of Figure legends, statistical comparisons between 2 groups was performed using the student’s *t* test with significance determined by 2 tailed *P* value < 0.05. For comparisons across more than 2 groups, -way ANOVA with Tukey’s multiple comparisons testing was used with *P <* 0.05 used to determine statistical significance, as indicated in each figure legend.

### Study approval.

Maintenance, editing, and directed differentiation of all cell lines was performed under regulatory approval of the Boston University Institutional Review Board (IRB; protocol H-33122). For derivation of ABCA3 mutant patient-specific iPSC lines, dermal fibroblasts from each individual were received from Washington University School of Medicine after review and approval by the Human Research Protection Office of Washington University School of Medicine.

### Data availability.

A Supporting Data Values file with all reported data values is available as part of the supplemental material.

## Author contributions

YLS, EEH, and DNK designed the project, performed experiments, and wrote the manuscript. HH performed the lipidomic analyses. PY performed Western blots. JAW and FSC provided patient tissue, clinical information, analyzed data, and edited the manuscript. CVM performed RNA-seq analyses. JK and AE performed co-IP/MS experiment. MJ performed iPSC reprogramming and archiving. KG performed cell differentiation and CFE measurements. All authors reviewed and approved the final manuscript.

## Supplementary Material

Supplemental data

Supplemental table 1

Supporting data values

## Version 1 01/16/2024

Electronic publication
