# Supplementary material for: Human pluripotent stem cell modeling of alveolar type 2 cell dysfunction caused by ABCA3 mutations
Source: J Clin Invest. 2024 Jan 16;134(2):e164274. doi: 10.1172/JCI164274 (PMC10786693; doi:10.1172/JCI164274)
Supplement: Supplemental data [file jci-134-164274-s131.pdf]

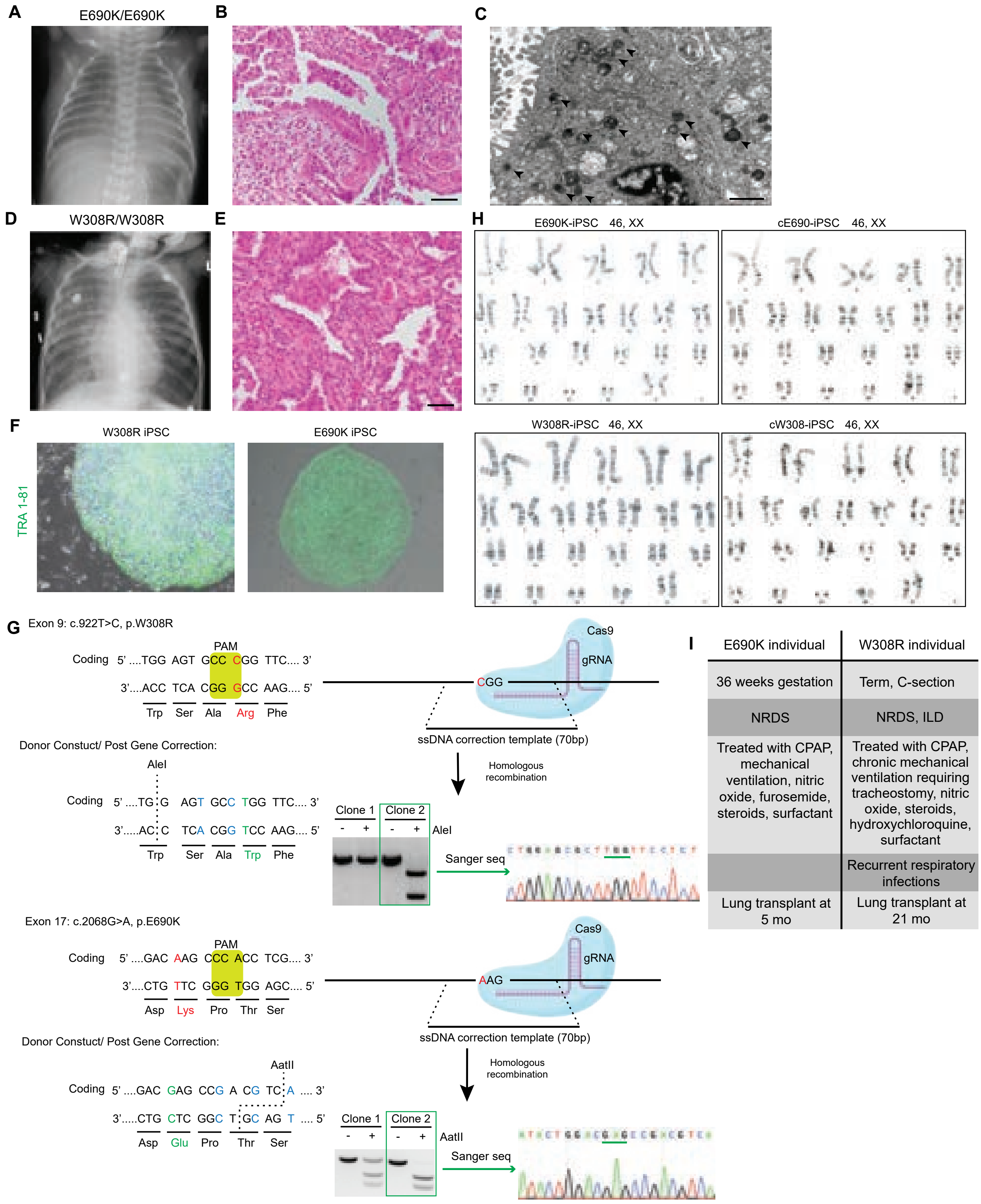

Figure S1 Clinical records and generation of E690K and W308R ABCA3 mutant patient iPSCs and syngeneic controls
(A) Chest x-ray of patient carrying homozygous E690K ABCA3 mutations showing bilateral pulmonary infiltrates.
(B) H&E staining of lung explant from the patient in panel (A), showing extensive alveolar remodeling, type II cell hyperplasia, interstitial thickening, lymphoid aggregates, and neutrophilic infiltrates. Scale bars, 100µm left, 25µm right. Airway (AW).
(C) TEM images of tissue from (B), showing irregular, small LBs (black arrow heads). Scale bars, 1µm.
(D) Chest x-ray of patient carrying homozygous W308R ABCA3 mutations, showing bilateral pulmonary infiltrates.
(E) H&E staining of explant from patient in (D), showing diffuse type II cell hyperplasia, intraalveolar macrophages, alveolar septal widening with interstitial fibrosis. Scale bars, 50µm left, 25µm right.
(F) TRA 1-81 pluripotency marker immunostains (green) of reprogrammed E690K and W308R iPSC colonies.
(G) CRISPR-Cas9 bi-allelic gene correction strategy for homozygous W308R (top) and E690K (bottom) mutations (red nucleotides). To facilitate screening of corrected clones, silent mutations (blue nucleotides) were introduced in the donor ssDNA correction template; successful homologous recombination was screened using restriction enzyme (AleI, AatII) digest, followed by Sanger sequencing confirmation.
(H) G-banding karyotypes of patient iPSC lines pre- and post- CRISPR-Cas9 gene correction.
(I) Clinical course from birth to lung transplantation in E690K and W308R ABCA3 mutant individual, also see supplemental methods.
NRDS= Neonatal respiratory distress syndrome; ILD= interstitial lung disease.

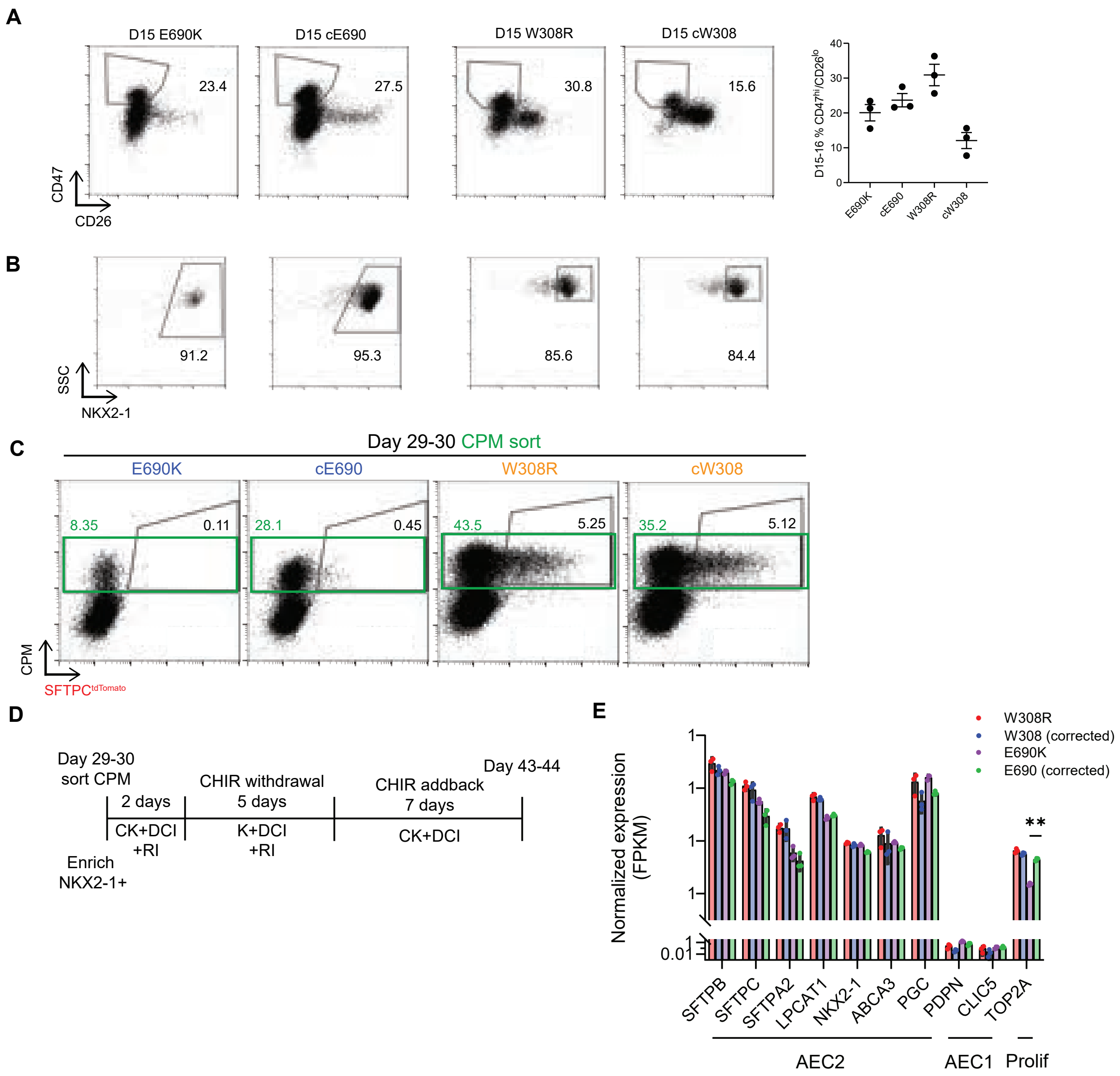

Figure S2 Step-wise directed differentiation and sorting to enrich NKX2-1+ distal lung epithelial progenitor cells during early stages of differentiation of patient-specific iPSCs (see also figure 1).

(A) Representative flow cytometry analyses of day 15 CD47^{hi}/CD26^{lo} cell sorting in all patient lines showing gates used for cell sorting to enrich for NKX2-1 expressing cells, according to methods detailed in Jacob et al. 2019. Biological triplicate separated at day 0, n=3. Bars, mean ± SE.

(B) Representative NKX2-1 intracellular staining analyzed by flow cytometry, showing enrichment of NKX2-1 expressing cells in all patient lines following CD47^{hi}/CD26^{lo} sorting. Dot plots are representative of triplicates samples.

(C) Representative flow cytometry analyses of day 29-30 CPM sorts to enrich for NKX2-1+ distal lung epithelial cells in all patient lines, showing percentages of cells expressing CPM (green gate) and SFTPC^{tdTomato} reporter (black gate).

(D) Timeline of CPM sort and CHIR withdrawal and addback from day 29-30 to day 43-44 of distal lung differentiation to enhance expression of the SFTPC^{tdTomato} reporter.

(E) Graph showing normalized expression of AEC2, AEC1, and proliferative markers in W308R and E690K mutant and corrected iAEC2s. Measured in fragments per kilobase of transcript per million mapped reads (FPKM) from bulk RNA sequencing; n=3. NB: TOP2A is the only significantly differentially expressed transcript shown, comparing mutant to corrected iAEC2s. **FDR adjusted p-value ≤0.01.

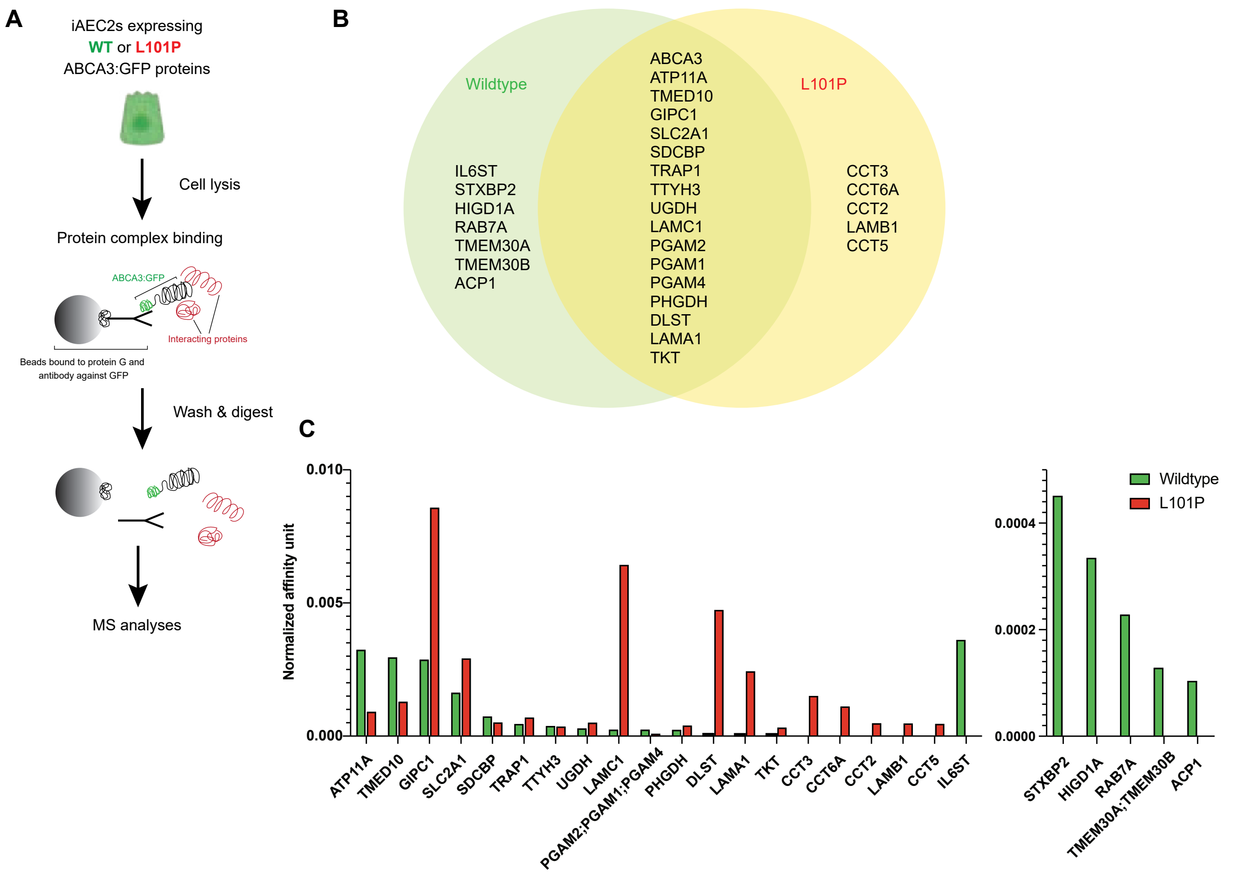

Figure S3 Mass spectrometry analyses of co-immunoprecipitated protein extracts from iAEC2s expressing wildtype or L101P mutant ABCA3:GFP proteins reveal unique, mutant-specific vs wildtype-specific candidate protein-protein interactions

(A) Schematic showing lysis of iAEC2s followed by co-immunoprecipitation (co-IP) followed by mass spectrometry (MS) analyses to identify proteins potentially interacting with wildtype and L101P ABCA3:GFP fusion proteins.

(B) Venn diagram showing proteins identified as potentially interacting with either wildtype or L101P mutant or both ABCA3:GFP fusion proteins.

(C) Bar graph showing affinity unit of shared and genotype-specific interacting protein partners to wildtype or L101P ABCA3:GFP fusion proteins. Normalized to the level of ABCA3 peptides in each co-IP/MS preparation.

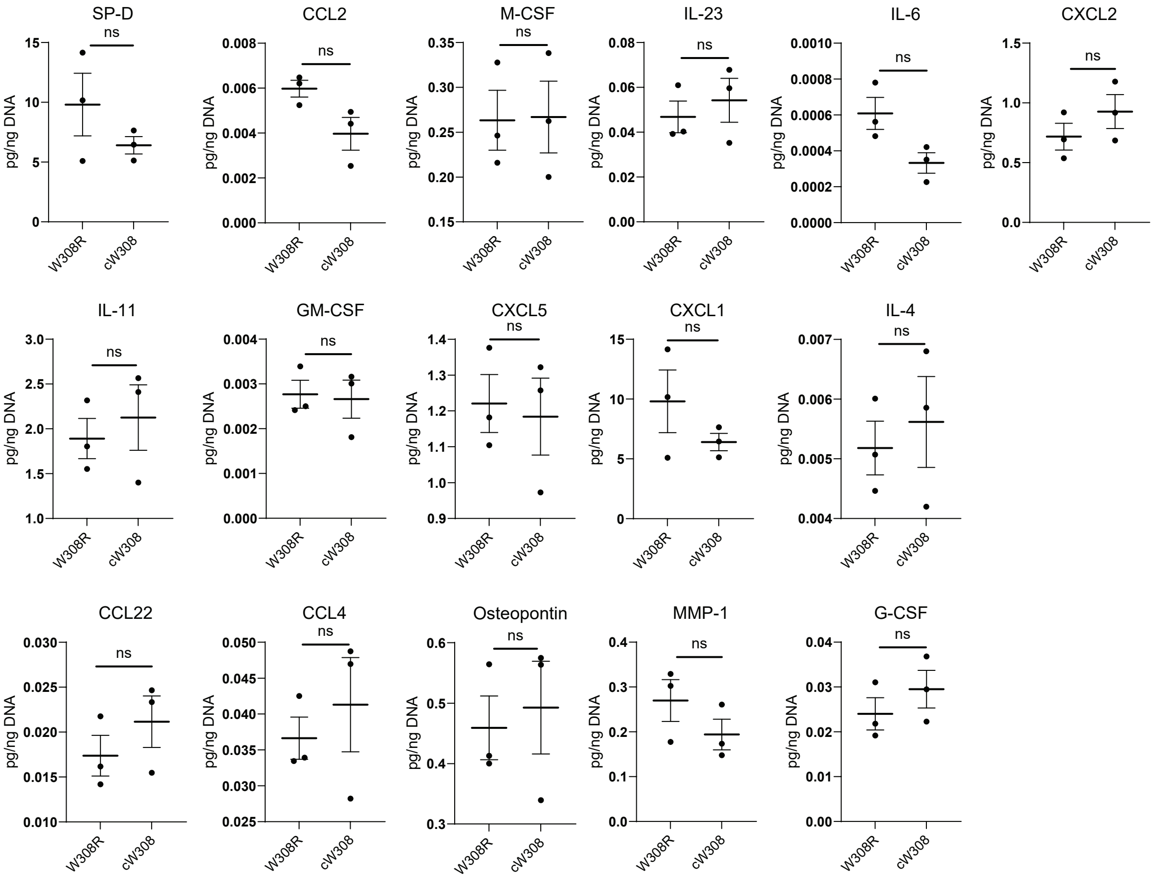

Figure S4 Measurement of cytokines and chemokines in supernatants of patient iAEC2s
Levels of indicated cytokines and chemokines released in the culture supernatants of 2D mono-layer cultured patient iAEC2s homozygous for the W308R mutation compared to corrected (cW308). Biological replicates (n=3), separated at day 0. Bars represent mean ± SE. **p≤ 0.01, two-tailed Student's t-test.

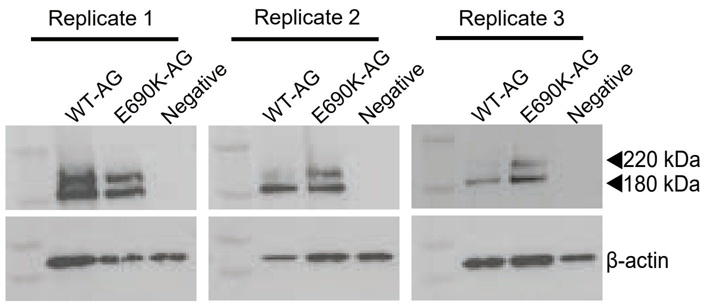

Figure S5 Processing of ABCA3 WT and mutant proteins in human iAEC2s
Western blots using an antibody against GFP to compare protein processing of ABCA3:GFP WT vs. E690K in iAEC2s. Negative controls are iAEC2s without the ABCA3:GFP fusion protein.

SUPPLEMENTAL METHODS:

Clinical, radiographic, and histologic phenotyping of donors with chILD

One patient with homozygous E690K and one patient with homozygous W308R *ABCA3* mutations each presented with rapid onset of neonatal respiratory symptoms which were refractory to all treatments. The patient with homozygous E690K *ABCA3* mutation (c.2068G>A) was a female infant born at 36 weeks gestation. Further genetic analysis revealed her homozygous *ABCA3* mutation to be the result of uniparental disomy of the maternal mutant allele. Within a few hours of birth, the patient developed symptoms of respiratory distress with chest radiographs revealing persistent bilateral pulmonary infiltrates (Fig S1A). An echocardiogram demonstrated tricuspid regurgitation and signs of pulmonary hypertension. Treatment for her respiratory symptoms with mechanical ventilation, supplemental oxygen, nitric oxide, steroids, and multiple doses of exogenous surfactant were ineffective, resulting in persistent respiratory failure. The patient received a bilateral lung transplant at 5 months of age. Explant histology demonstrated diffuse interstitial fibrosis, extensive alveolar remodeling with cystic spaces, diffuse AEC2 hyperplasia, extensive neutrophilic infiltrates of the airways and alveoli, chronic interstitial inflammation with lymphoid aggregates, and intimal thickening of small pulmonary arteries (Fig 1A, S1A, B). Electron microscopy of AEC2s demonstrated small, dense lamellar bodies and tightly wound, small lamellar structures (Fig S1C). Genetic evaluation found no mutations in other genes associated with surfactant production, such as SFTPC or SFTPB genes.

The patient identified with homozygous W308R ABCA3 mutation (c.922T>C) was a term female infant who developed progressive tachypnea, increased work of breathing, and respiratory failure requiring mechanical ventilation. A chest CT taken at 1 month of age demonstrated diffuse, severe, bilateral interstitial infiltrates, ground glass opacities and scattered small cystic areas (Fig S1D). Treatment with exogeneous surfactant, supplemental oxygen, steroids, and hydroxychloroquine, did not result in clear improvement in lung function. Due to persistent respiratory failure and recurrent infections from her underlying lung disease, at 21 months of age the patient underwent lung transplantation. Explant histology demonstrated extensive alveolar remodeling diffuse AEC2 hyperplasia, intra-alveolar macrophages, alveolar septal thickening and interstitial fibrosis (Fig 1A, S1E).

Patient iPSCs and reprogramming and maintenance

All human iPSCs were maintained in feeder free conditions, cultured on Matrigel-coated (Corning, 354277) plates in mTeSR media (StemCell Technologies, 85850), and passaged using Gentle Cell Dissociation Reagent (StemCell Technologies, 100-0485). Reprogramming of the BU3 human iPSC line was previously reported in Kurmann et al. (1) and editing of this line to target an ABCA3:GFP fusion cassette to the endogenous ABCA3 locus (BU3-AG) was previously reported (2). Maintenance, editing, and directed differentiation of these lines was performed under regulatory approval of the Boston University Institutional Review Board (IRB; protocol H-33122) with donor informed consent. For derivation of ABCA3 mutant patient-specific iPSC lines, patient tissue

samples were received from Washington University School of Medicine after review and approval by the Human Research Protection Office of Washington University School of Medicine. Genetic evaluation found no mutations in other genes associated with surfactant production, such as SFTPC or SFTPB genes. Reprogramming of dermal fibroblasts from the patient with homozygous E690K *ABAC3* was performed using the excisable floxed STEMCCA lentiviral vector, followed by vector excision with transient Cre transfection as we have previously published (3). Clone "ABCA31" (alias ABCA3 SP300) was selected for this project. Dermal fibroblasts from the patient with W308R *ABCA3* was performed with the Sendai virus Cytotune 2 Kit (Thermo Fisher, A16517) according to the manufacturer's instructions. Clone "ABCA35" (alias ABCA3 W308R) was selected for this project. Picked candidate clones from each genotype were characterized for pluripotency by staining for pluripotency markers using monoclonal mouse antibodies against TRA1-81 and TRA 1-60 (Stem Cell Technologies, Catalog # 60065AD.1, Catalog # 60064AD.1) and found to be karyotypically normal (Cell Line Genetics; Figure S1). All iPSC lines produced are catalogued and can be requested through the Center for Regenerative Medicine (CReM) of Boston University and Boston Medical Center, via their iPSC Core and Lung Disease Specific Biorepository: www.crem.bu.edu.

Gene Editing of Human iPSC Lines

E690K and W308R ABCA3 mutant patient-derived iPSC lines were mono-allelically targeted with a tdTomato reporter at the ATG of the endogenous SFTPC locus using TALENS gene editing tools following the same methods detailed in Jacob et al. (4).

<u>CRISPR-Cas9 gene editing of the ABCA3 locus to correct or knock-in mutations:</u>

For bi-allelic, foot-print free ABCA3 gene correction of patient iPSC lines, a 20bp guide RNA (gRNA) specific to either the E690K mutation 5'-GATGGCGTCCATGCCCGAGG-3' or the W308R mutation 5'-GCTGGCTGCACTGGAGTGCCCGG-3' was designed using crispr.mit.edu, based on proximity to mutation site and fidelity score. Each gRNA was inserted using conventional cloning techniques into pSpCas9 plasmids (5) containing sequences encoding the Cas9 protein and GFP. For homologous recombination after CRISPR Cas9-induced double stranded break, 70 bp single stranded DNA oligonucleotide (ssODN) donors were designed for gene-correction for the E690K mutation (5' ACTGTGTCTCTCCCTCCAGGTGCTGATACTGGAC GAG CCG ACG TCA GGC ATG GAC GCC AT C TCCAGGAGG 3') and the W308R mutation (5' GGCGATGAGGAGGAAGAGGAAGAACAAGAGGAACCAAGCGCTCCAGTGCAGCCA GCTGCTGAGCCCCATC 3'). Each donor ssODN contains silent mutations resulting in DNA sequences recognized by restriction enzymes (AatII site for E690K, and AleI for W308R) upon successful homologous recombination, allowing for screening of iPSC clones with restriction digestion of PCR products.

iPSC nucleofection of plasmids and ssODNs was performed using the P3 Primary Cell 4D-Nucleofector X Kit (Lonza, cat no. V4XP-3024). $5X10^6$ iPSCs were nucleofected with 5μg of pSpCas9 plasmid containing desired gRNA and 5μg of ssODN in 100 μl total volume of P3 nucleofection solution (Lonza) and re-plated on 4 wells of matrigel coated 6-well tissue culture dish (Corning), fed with mTeSR media (Stem Cell Technologies) and rock inhibitor (RI), then re-fed with just mTeSR after 24 hours. 48 hours after nucleofection,

GFP+ cells were sorted and plated at a density of 1,000-3,000 cells per well of a 6 well tissue culture plated pre-seeded with mouse embryonic fibroblast (MEF) feeder cells. After approximately 2 weeks, clonal outgrowths were harvested for genomic DNA for PCR screening using primer pairs designed to surround the region of homologous recombination, F: 5' AAGGGCCTGTCACGTCAGAA 3', R: 5' CTGATCTGAGGGCCCTTCATGAA 3' for the E690K locus, and primer pairs, F: 5' CAGGCGCTTTTGGTCAGTGAA 3', R: 5' CTACATTTGGCTTCACCTGCAGG 3', for the W308R locus. PCR amplicons were screened using restriction enzyme digestion as shown in the supplemental figures. Gene-correction of successfully digested clones was confirmed by Sanger sequencing.

For CRISPR Cas9 single nucleotide E690K and W308R mutagenesis of the wildtype $BU3^{ABCA3:GFP}$ iPSC line, the same guide RNAs used for gene-correction of patient-specific iPSC lines were used in conjunction with new ssODN donors: 5' ACTGTGTCTCTCCCTCCAGGTGCTGATACTGGACAAGCCCACCTCGGGCATGGAC GCCATCTCCAGGAGG 3' for E690K mutation, and 5' GGCGATGAGGAGGAAGAGGAAGAACAAGAGGAACCGAGCGCTCCAGTGCAGCCA GCTGCTGAGCCCCATC 3' for W308R mutation containing mutated sequences.

For the L101P mutagenesis of $BU3^{ABCA3:GFP}$ iPSC line, guide RNA 5' CGTCACTGAGACAGTGCGCAGGG 3' and ssODN of 5' TGTCTCACCTCGCATGTTGATCACAGGTGCTCTGCGCACTGTCTCAGTGACGGTCT TGGCAGCGTCACTG 3' was used, containing restriction digest sequence for BsiHKAI restriction enzyme for recombination screening of targeted clones.

Directed Differentiation and Maintenance of iAEC2s

Directed differentiation of iAEC2s were performed as detailed in our previously published protocol (4, 6). In brief, day 0 PSCs were differentiated into definitive endoderm (day 0-3) using StemDiff Endoderm Kit (Stem Cell Technologies, 05110), followed by anterior foregut endoderm (day 3-6) using DS/SB media (2uM dorsomorphin, Stemgent, 040024; 10μM SB431543, Biotechne, 1614), then further specified into NKX2-1+ lung epithelial progenitors using CBRa media (3μM CHIR99021, Biotechne, 4423; 10ng/ml rhBMP4, BioTechne, 314BP; 100nM retinoic acid, Sigma-Aldrich, R2625; day 6-15). On day 15, NKX2-1 expressing lung epithelial progenitors were sorted either by $NKX2\text{-}1^{GFP}$(BU3-NGST line) or using $CD47^{hi}/CD26^{lo}$ sorting to enrich for NKX2-1+ cells (7). Sorted cells were plated in 3D Matrigel cultures and fed with distalizing CK+DCI media (3μM CHIR99021, 10ng/ml KGF, 50nM dexamethasone, 0.1mM cyclic AMP and 0.1mM IBMX), as detailed in Jacob et al. (6).

Additionally, to increase the frequencies of $SFTPC^{tdTomato}$ or ABCA3:GFP-expressing iAEC2s, CHIR "withdrawal and addback" to distal lung progenitor cells was conducted as previously published in Jacob et al. (6), first by plating day 30 CPM sorted cells in 3D matrigel and feeding with CK+DCI and RI for 48 hours, followed by re-feeding with KGF+ DCI and RI (KDCI+RI; i.e. "CHIR withdrawal") for 5 days, followed by re-feeding with the standard CK+DCI media for the duration of the experiment indicated in the text..

2D Monolayer Culture of iAEC2s

2D monolayered iAEC2 culture were made by plating either day 15 CD47hi/CD26lo sorted lung progenitors, or day 43+ alveolospheres, after treating with trypsin to prepare a single-cell suspension. Single cell suspensions were then plated on Matrigel-coated 48-well tissue culture plates (Corning) at 300,000 to 600,000 cells per well. 2D cultures were fed with CK+DCI with RI every other day until confluent.

Quantification of intracellular vesicle/lamellar body size by ABCA3:GFP fluorescence microscopy

For measurements of ABCA3:GFP+ vesicles in wildtype and *ABCA3* mutant iAEC2s, 50 representative vesicles were measured using ImageJ software measuring tool across three separate images per genotype and across 8-10 cells. Measurement of A549 cells expressing wildtype or mutant ABCA3:GFP+ vesicles was performed on confocal microscopy images using the NIS-Elements software (Nikon).

Quantitative RT-qPCR

qRT-PCR measuring expression levels of key AEC2 and non-lung endodermal genes was performed as we detailed previously in Hawkins et al. (7). Briefly, RNA was harvested following the manufacturer's instructions using Qiazol and miRNeasy mini kits (QIAGEN). cDNA was generated by reverse transcription of 100ng RNA from each sample using Applied Biosystems High-Capacity cDNA Reverse Transcription Kit. For qPCR, either 20μl reactions (for use in Applied Biosystems StepOne 96-well System) or 12 μl reactions (for use in Applied Biosystems QuantStudio7 384-well System) were prepared using 2μl of diluted cDNA and run for 40 cycles. All primers were TaqMan probes from Applied Biosystems (specific primer cat. no. referenced in Jacob et al. (4)). Relative expression was calculated using average cycle value (Ct) of samples normalized to 18S control and reported as fold change ($2^{-\Delta\Delta Ct}$), with fold change of 1 assigned to day 0 undifferentiated PSCs, unless otherwise indicated in the text. For genes that were undetectable after 40 cycles of PCR, a Ct value=40 was assigned to allow fold change calculations.

Surfactant secretion after secretagogue stimulation of iAEC2s as measured by fluorescence microscopy and mass spectrometry

For visualization of phospholipid secretion using day 75 BU-AG iAEC2s, day 72 cultured ABCA3:GFP+ iAEC2s were single-cell dissociated and plated as a 2D monolayer on Matrigel-coated coverslip-bottomed dishes (MatTek, part no. P35G-1.5-14-C) at $1X10^6$ cells per well. iAEC2 secretion was induced using a secretagogue cocktail consisting of final concentrations of 100nM ATP (Thermo Fisher cat no. R0441) and 300nM Phorbol12-myristate 13-acetate (PMA, Cayman Chemicals, item no. 10008014). Visualization of secreted lipid contents was achieved by feeding cultured cells with 5μg/mL of FM4-64 dye (Thermo Fisher, cat no. T13320) 20 min prior to induction with or without secretagogues.

For lipidomic analyses of patient iAEC2 supernatants, day 171-173 patient iAEC2s from triplicate differentiations (n=3, separated from day 0 on for E690K vs cE690 syngeneic pairs) and separated at day 133 on (for W308R vs cW308 syngeneic pairs) were plated in 2D monolayer cultures at 300,000 cells per well and fed with CK+DCI+RI for 14 days.

On day 185-187, cells were treated either with the indicated secretagogues or DMSO vehicle control for 24 hours followed by collection of cell pellets for DNA quantitation and culture supernatants for lipidomic analyses by mass spectrometry as detailed in Jacob et al. (4). Total phosphatidylcholine (PC) composition and 32:0 PC (dipalmitoylphosphatidylcholine; DPPC) composition was reported as "Absolute Quantitation" nmol/μg protein or nmol/μg DNA.

Immunofluorescence Imaging

Routine live-cell fluorescence imaging was done using a Keyence BZ-X800 microscope (Keyence, Japan). Live-cell confocal imaging of secretagogue-induced iAEC2 secretion and other 2D plated iAEC2s was conducted using reagents described above and an LSM 880 Laser Scanning Confocal Microscope (Zeiss, Germany).

Flow Cytometry and Cell sorting

Preparation of single-cell suspension for flow cytometry and cell sorting was described previously (4, 6). Prepared samples were either directly sorted based on fluorescence reporter expression (ABCA3:GFP, $SFTPC^{tdTomato}$, both, or neither) on a MoFlo Astrios Cell Sorter (Beckman Coulter) or stained with primary and secondary antibodies as indicated in the text prior to cell sorting. For day 15 enrichment of NKX2-1+ primordial lung progenitors, cell surface antigen staining for $CD47^{hi}/CD26^{lo}$ cell population was performed using methods previously published in Hawkins et al. (7). For day 30 re-enrichment of NKX2-1+ lung epithelial population, staining of cells in single-cell suspension for CPM for flow cytometry and cell sorting were done at 4°C using primary mouse monoclonal antibodies against human CPM (1:200, Fujifilm Wako, 014-27501) for 30min followed by staining with secondary Alexa Fluor 647 conjugated antibody (1:500, ThermoFisher Scientific, A32787) for another 20 min.

ABCA3:GFP Co-immunoprecipitation and Mass Spectrometry analyses

To identify potential protein binding partners for ABCA3, we prepared iAEC2 cell pellets as indicated in the text. These pellets were lysed in buffer containing 30 mM Tris-HCl, 150 mM NaCl, 1% N-dodecylmaltoside, and complete protease and phosphatase inhibitors (Roche) followed by 1 freeze thaw cycle and sonication at 10%, 15s, 3s pulse. Supernatants were collected after centrifugation at 14,000g for 30 min at 4°C. Immunoprecipitation was performed with anti-GFP (Invitrogen GF28R) or IgG control and Protein G Dynabeads (Invitrogen), 3 hour incubation at 4°C. Protein complex bound beads were washed twice using Lysis buffer without detergent and once with 100 mM triethylammonium bicarbonate. On bead Trypsin digestion was performed with 750ng of trypsin (Pierce) in 100 mM triethylammonium bicarbonate overnight at 37°C. Peptides were desalted using a C18 ZipTip (Millipore) and subjected to reverse-phase LC separation on a 60-min gradient and analyzed on a Q Exactive HF-X (Thermo Fisher Scientific). Data-dependent fragmentation used collision-induced dissociation. RAW files were searched using MaxQuant under standard settings using the UniProt human database, allowing for two missed trypsin cleavage sites, variable modifications for N-terminal acetylation, and methionine oxidation. Candidate peptides and protein identifications were filtered on the basis of a 1% false discovery rate threshold based on

searching of the reverse sequence database. To remove potential contaminants, we eliminated proteins detected in IgG control group. Comparison and analyses of potential protein interacting partners between wildtype and L101P ABCA3:GFP mutant fusion protein were performed by normalizing the intensity level of ABCA3 peptides within each of the wildtype and L101P precipitate samples.

Bulk RNA sequencing

The following samples were harvested in Qiazol (QIAGEN) for bulk RNA-sequencing analysis: (1) day 43 $SFTPC^{tdTomato}$+ E690K patient iAEC2; (2) day 43 $SFTPC^{tdTomato}$+ cE690 patient iAEC2; (3) day 44 $SFTPC^{tdTomato}$+ W308R patient iAEC2s; (4) day 44 $SFTPC^{tdTomato}$+ cW308 patient iAEC2s; (5) day 43 ABCA3:GFP+ wildtype BU3 iAEC2s; (6) day 44 E690K ABCA3:GFP+ BU3 iAEC2s; (7) day 44 W308R ABCA3:GFP+ BU3 iAEC2s. Triplicate differentiations of each line were performed (n=3; separated from day 0). RNA extractions, library preparations, and bioinformatic analyses were performed using methods we have previously published (2). The triplicated mutant vs normal control differentiations were prepared and sequenced head-to-head to avoid any potential technical batch artifacts. Briefly, sequencing libraries were prepared from total RNA extracted from each indicated sample using Illumina TruSeq RNA Sample Preparation Kit v2. mRNA was isolated using magnetic bead-based poly(A) selection, fragmented, and randomly fragmented for reverse transcription, followed by synthesis of cDNA fragments. cDNA fragments were then end-paired and ligated to Illumina Paired-End sequencing adapters. The products were end-paired and PCR-amplified to create the final cDNA library. Libraries were sequenced on an Illumina NextSeq 500 to generate an average of 49 million paired-end reads per sample for the patient-specific samples. The sequencing of the 3 libraries from BU3 iPSC-derived iAEC2s generated an average of 53 million paired-end reads per sample. The quality of the raw data was assessed using FastQC v.0.11.7. Sequence reads were aligned to a combination of the human genome reference (GRCh38) and GFP reporter sequence, using STAR v.2.5.2b (8). Counts per gene were summarized using the featureCounts function from the subread package v.1.6.2. The edgeR package v.3.25.10 was used to import, organize, filter and normalize the data. Genes that were not expressed in at least one of the experimental groups were filtered out (keeping only genes that had at least 0.5 counts per million of mapped reads in at least 3 libraries). The TMM method was used for normalization. Principal Component Analysis (PCA) and Multidimensional Scaling (MDS) were used for exploratory analysis and to assess sample similarities. Differentially expressed genes (DEGs) between samples were identified by using the limma package v.3.52.0 and its voom method for fitting linear models, doing empirical Bayes moderation to estimate gene-wise variability, and finally, testing significance based on the moderated t-statistic. All DEGs, false discovery rate (FDR)-adjusted p values, and gene expression fold change values are listed in the supplemental tables (tables S1A, S1B, and S2). Gene set analysis was performed using the GSEA package (9). All sequencing datasets have been deposited with the online Gene Expression Omnibus (see main methods section for accession numbers).

EdU incorporation and colony forming efficiency assays

For colony formation efficiency (CFE) assays, triplicate differentiations (n=3, separated at day 0) of patient ABCA3 mutant or gene-corrected day 43 $SFTPC^{tdTomato}$-sorted iAEC2s were plated at 400 cells/μl in 25μl 3D matrigel droplet. Stitched, and full focused, Z-stacked bright field images were taken 10 days after cell plating using BZ-X800 Keyence microscope for CFE analyses reported as total number of colonies divided by input cell per droplet. For EdU incorporation assay of $SFTPC^{tdTomato}$+ iAEC2s, the same cells used for CFE quantitation were inoculated on day 10 with 10μM EdU for 24 hours followed by cell sorting for SFTPCtdTomato+ iAEC2s. Sorted cells were fixed, permeabilized, and stained with the Click-iT reaction mixture (Invitrogen) according to the manufacturer's protocol. The percentage of iAEC2s that incorporated EdU was determined by flow cytometry (Stratedigm, CA, USA).

Measurement of NFκB Pathway Activity in Patient iAEC2s

Bioluminescence quantification of p50/65 heterodimer binding activity was performed in AEC2s using our published lentiviral NFkB signaling reporter vector (10) with methods for transduction of iAEC2s, sorting, and bioluminescence measurements detailed in our prior publication (11). Briefly, day 258 W308R and cW308 patient iAEC2s, and day 158 E690K and cE690 iAEC2s grown in 3D culture were dissociated to single-cell suspension for lentiviral infection. 100,000 iAEC2s from each genotype were infected with the lentivirus at 20 MOI and polybrene for 4 hours at 37 ^{o}C in 1.5ml tube and, plated in triplicate 3D matrigel droplets and re-fed with CK+DCI. 14 days after plating, 15,000 GFP+ infected cells and uninfected cells per genotype were added to 96-well microplates (Thermo Fisher cat. no. M33089) for luminescence measurements using Dual-Luciferase Reporter Assay Kit (Promega) according to manufacturer's recommendations and using Infinite 200 PRO microplate reader (TECAN, Switzerland).

iAEC2 Supernatant Cytokine and Chemokine Measurements

Patient iAEC2 supernatants were collected from triplicate differentiations (all lines separated at day 0, n=3) of day 97 E690K and cE690 patient iAEC2s and day 100 W308R and cW308 patient iAEC2s grown in 2D monolayered culture 8 days after 2D plating on matrigel coated 48-well plates. Supernatant protein concentrations of SP-D, M-CSF, IL-23, GM-CSF, CXCL5, CXCL1, CXCL17, CCL20, CCL11, CCL17, CCL22, CCL4, OPN, MMP-1, MMP7, MMP-10, MMP-13, IL-8, IL-1beta, TNF-alpha, IL-11, IL-13, IL-33, IL-6, IL-4, G-CSF, CXCL1, CXCL2, CX3CL1, CCL2, IFN-alpha, IFN-gamma, IFN-beta, were measured by using human magnetic Luminex assay (R&D systems) on Bio-Plex 200 multiplexing analyzer system (Bio-Rad).

1. Kurmann AA, Serra M, Hawkins F, Rankin SA, Mori M, Astapova I, et al. Regeneration of Thyroid Function by Transplantation of Differentiated Pluripotent Stem Cells. *Cell Stem Cell.* 2015;17(5):527-42.
2. Sun YL, Hurley K, Villacorta-Martin C, Huang J, Hinds A, Gopalan K, et al. Heterogeneity in Human Induced Pluripotent Stem Cell-derived Alveolar Epithelial Type II Cells Revealed with ABCA3/SFTPC Reporters. *Am J Respir Cell Mol Biol.* 2021;65(4):442-60.

3. Somers A, Jean JC, Sommer CA, Omari A, Ford CC, Mills JA, et al. Generation of transgene-free lung disease-specific human induced pluripotent stem cells using a single excisable lentiviral stem cell cassette. *Stem Cells.* 2010;28(10):1728-40.
4. Jacob A, Morley M, Hawkins F, McCauley KB, Jean JC, Heins H, et al. Differentiation of Human Pluripotent Stem Cells into Functional Lung Alveolar Epithelial Cells. *Cell Stem Cell.* 2017;21(4):472-88 e10.
5. Ran FA, Hsu PD, Wright J, Agarwala V, Scott DA, and Zhang F. Genome engineering using the CRISPR-Cas9 system. *Nat Protoc.* 2013;8(11):2281-308.
6. Jacob A, Vedaie M, Roberts DA, Thomas DC, Villacorta-Martin C, Alysandratos KD, et al. Derivation of self-renewing lung alveolar epithelial type II cells from human pluripotent stem cells. *Nat Protoc.* 2019;14(12):3303-32.
7. Hawkins F, Kramer P, Jacob A, Driver I, Thomas DC, McCauley KB, et al. Prospective isolation of NKX2-1-expressing human lung progenitors derived from pluripotent stem cells. *J Clin Invest.* 2017;127(6):2277-94.
8. Dobin A, Davis CA, Schlesinger F, Drenkow J, Zaleski C, Jha S, et al. STAR: ultrafast universal RNA-seq aligner. *Bioinformatics.* 2013;29(1):15-21.
9. Subramanian A, Tamayo P, Mootha VK, Mukherjee S, Ebert BL, Gillette MA, et al. Gene set enrichment analysis: a knowledge-based approach for interpreting genome-wide expression profiles. *Proc Natl Acad Sci U S A.* 2005;102(43):15545-50.
10. Wilson AA, Kwok LW, Porter EL, Payne JG, McElroy GS, Ohle SJ, et al. Lentiviral delivery of RNAi for in vivo lineage-specific modulation of gene expression in mouse lung macrophages. *Mol Ther.* 2013;21(4):825-33.
11. Alysandratos KD, Russo SJ, Petcherski A, Taddeo EP, Acin-Perez R, Villacorta-Martin C, et al. Patient-specific iPSCs carrying an SFTPC mutation reveal the intrinsic alveolar epithelial dysfunction at the inception of interstitial lung disease. *Cell Rep.* 2021;36(9):109636.
